# Butt-seq: a new method for facile profiling of transcription

**DOI:** 10.1101/gad.350434.123

**Published:** 2023-05-01

**Authors:** Albert D. Yu, Michael Rosbash

**Affiliations:** Department of Biology, Howard Hughes Medical Institute, National Center for Behavioral Genomics, Brandeis University, Waltham, Massachusetts 02453, USA

**Keywords:** circadian rhythms, nascent RNA, RNA polymerase II pausing, superelongation complex, transcriptional profiling, transcriptional regulation

## Abstract

A wide range of sequencing methods has been developed to assess nascent RNA transcription and resolve the single-nucleotide position of RNA polymerase genome-wide. These techniques are often burdened with high input material requirements and lengthy protocols. We leveraged the template-switching properties of thermostable group II intron reverse transcriptase (TGIRT) and developed Butt-seq (bulk analysis of nascent transcript termini sequencing), which can produce libraries from purified nascent RNA in 6 h and from as few as 10,000 cells—an improvement of at least 10-fold over existing techniques. Butt-seq shows that inhibition of the superelongation complex (SEC) causes promoter-proximal pausing to move upstream in a fashion correlated with subnucleosomal fragments. To address transcriptional regulation in a tissue, Butt-seq was used to measure the circadian regulation of transcription from fly heads. All the results indicate that Butt-seq is a simple and powerful technique to analyze transcription at a high level of resolution.

## Abstract (precis)

In this study, Yu and Rosbash describe a new methodology, Butt-seq, for the bulk sequencing analysis of nascent transcript termini. They demonstrate how Butt-seq can be used to understand transcriptional dynamics such as RNAPII pausing and circadian gene regulation.

Although the analysis of messenger RNA (mRNA) can usually be done with simple RNA-seq methods, the analysis of transcription and nascent RNA is faced with greater challenges. Not only is nascent RNA less abundant, but it is also substantially more heterogeneous than mRNA; nascent RNA contains diverse transcripts beyond pre-mRNA, and these transcripts are in various states of active or stalled synthesis. This complexity requires careful consideration.

There are three general classes of techniques that analyze transcription. The first, chromatin immunoprecipitation and sequencing (ChIP-seq), immunoprecipitates RNA polymerase II (RNAPII) and sequences the associated DNA fragments. Such a standard ChIP-seq approach provides a low-resolution view of RNAPII localization, although single-nucleotide positioning can be achieved with modifications such as ChIP-nexus (He et al. 2015). The second class, run-on techniques such as global run-on sequencing (GRO-seq) and precision run-on sequencing (PRO-seq), isolate nuclei containing RNA undergoing active synthesis. Transcription is then begun in vitro with tagged nucleotides that can be affinity-purified and sequenced (Core et al. 2008; Mahat et al. 2016). Although these run-on techniques feature the greatest selectivity for actively synthesized RNA, they cannot capture RNA in stalled synthesis complexes, such as those with backtracked RNAPII (Wissink et al. 2019). The third class of techniques relies on the biochemical purification of nascent RNA and therefore captures RNA in both active and stalled RNAPII complexes.

The remarkable stability of the RNA polymerase/nascent RNA/DNA ternary complex enables the isolation of nascent RNA with buffers containing a high concentration of salt and urea (Wuarin and Schibler 1994). Techniques leveraging biochemically purified nascent RNA include preparing libraries from the entire length of the nascent RNA molecule, such as with Nascent-seq or chromatin-associated RNA sequencing (chrRNA-seq) (Khodor et al. 2012; Sousa-Luís et al. 2021). Other techniques aim to track RNA synthesis at single-nucleotide resolution by ligating adapters to and sequencing the 3′ ends of nascent RNA molecules; the logic is that the 3′ end is the most recently synthesized base. These 3′ end techniques include short capped RNA sequencing (scRNA-seq), 3′ nucleotide sequencing (3NT-seq), and human native elongating transcript sequencing (hNET-seq) (Nechaev et al. 2010; Weber et al. 2014; Mayer et al. 2015). scRNA-seq and 3NT-seq also feature the enrichment of capped RNA by digestion of uncapped RNA with terminator exonuclease. A variation of 3′ end sequencing techniques involves the immunoprecipitation of RNAPII to enable phosphorylation state-specific profiling of nascent RNA. They include native elongating transcript sequencing (NET-seq) and its derivatives; e.g., mammalian NET-seq (mNET-seq) and enhanced NET-seq (eNET-seq) (Churchman and Weissman 2012; Nojima et al. 2015; Fong et al. 2022).

These powerful techniques that sequence biochemically purified nascent RNA are also confronted by some contamination issues; for example, by mature chromatin-associated RNAs such as snoRNAs. Thus, care must be taken when interpreting signal. Moreover, all of these methods share two less than ideal features: The protocols are relatively complex and time-consuming and have quite high input material requirements.

To circumvent these features, we have devised a novel approach toward sequencing the 3′ ends of biochemically purified nascent RNA, which we have named Butt-seq (bulk analysis of nascent transcript termini sequencing). Butt-seq leverages an optimized thermostable group II intron reverse transcriptase (TGIRT) reverse transcription protocol to couple adapter ligation and reverse transcription into a single step (Mohr et al. 2013).

TGIRT was the first among a growing class of group II intron reverse transcriptases that exhibits useful characteristics such as high processivity and/or thermal stability. These features have seen TGIRT used in a variety of biochemical assays, including sequencing of highly structured RNAs such as tRNAs and small RNAs, RNA structure probing, ssDNA sequencing, and others (Wu and Lambowitz 2017; Zubradt et al. 2017; Yao et al. 2020; Xu et al. 2021; Begik et al. 2023). TGIRT is also distinguished by its ability to template-switch from the 5′ end of a fully reverse-transcribed RNA template onto the 3′ end of a new RNA template. By using an RNA/DNA hybrid primer that mimics a fully reverse-transcribed RNA molecule, TGIRT can “ligate” the DNA half of the primer onto any subsequent cDNA by template-switching to a target RNA molecule (Mohr et al. 2013). Typical 3′ end-labeling techniques rely on T4 RNA ligase to ligate an adapter, an RNA fragmentation step, and cDNA synthesis conducted in sequential steps (Churchman and Weissman 2012).

This Butt-seq protocol is used for second as well as first strand synthesis with several other optimizations, such as a salt concentration-switching step to maximize the efficiency of the initial template switch and minimize subsequent, undesirable template switches; the protocol also uses a low concentration of dNTPs to minimize DNA fragment size. These features eliminate the need for RNA fragmentation and condense multiple materially demanding gel extraction steps into a single post-PCR extraction step. Butt-seq can prepare libraries from purified nascent RNA in as few as 6 h and exhibits reproducible signal with inputs as low as 10,000 *Drosophila* S2 cells. This input requirement represents at least a 10-fold improvement over existing protocols; other NET-seq-like techniques typically reference inputs of 1,000,000–10,000,000 cells, while qPRO-seq requires 100,000 cells (Churchman and Weissman 2012; Mayer et al. 2015; Nojima et al. 2015; Judd et al. 2020). Butt-seq also is effective with limited input material from biological tissue, which we demonstrate by profiling small numbers of *Drosophila* heads.

When selecting a technique to analyze nascent RNA, it is important to understand the limits of each technique. For example, 3′ end-labeling is a powerful approach for resolving RNA polymerase positioning, but an increase in signal density can reflect either an increase in transcription or a decrease in transcriptional elongation rate. Here we examined this ambiguity by using Butt-seq to investigate the regulation of core circadian genes. Circadian rhythms are governed by a transcriptional feedback loop wherein the clock (*Clk*)/cycle (*cyc*) heterodimer drives the transcription of negative regulator genes period (*per*) and timeless (*tim*), which repress the activity of *Clk/cyc* and thereby down-regulate their own transcription (Hardin 2005). This feedback loop is a well-characterized paradigm driven by de novo transcription, so we examined the transcription of core clock genes to assess the ability of Butt-seq to measure relative transcription rate (So and Rosbash 1997; Rodriguez et al. 2013).

Promoter-proximal pausing is another feature of transcription that we address with Butt-seq. Such pausing is a ubiquitous and conserved regulatory step in transcriptional regulation, wherein RNA polymerase pauses after transcribing a short stretch of RNA to await further regulatory signaling before proceeding into processive elongation (Jonkers and Lis 2015). Pause release is dependent on phosphorylation of the RNAPII C-terminal domain (CTD) by cyclin-dependent kinase 9 (Cdk9) as part of the positive transcription elongation factor b (P-TEFb) complex (Jonkers and Lis 2015; Liang et al. 2018). P-TEFb is largely sequestered in its inactive form, but its incorporation into the superelongation complex (SEC) is one means by which it can be directed to drive pause release. The *Drosophila* SEC is comprised of the elongation suppressor of Triplolethal [*Su(Tpl)*], ELL-associated factor (*Eaf*), ENL/AF9-like protein (*ear*), and the AFF (AF4/FMR2 family)-like protein Lilliputian (*lilli*) (Dahlberg et al. 2015). We treated S2 cells with the AFF4/*lilli* inhibitor KL-1 and examined the effect on pause release with Butt-seq. Pharmaceutically inhibiting the SEC with KL-1 causes pause sites to recede upstream in a chromatin-dependent fashion, showing that Butt-seq can resolve RNA polymerase localization at single-nucleotide resolution and implying that promoter-proximal pausing regulation may involve the clearance of multiple discrete pausing sites.

Altogether, we establish Butt-seq as a simple and powerful method capable of resolving multiple aspects of transcription that offers several attractive improvements over existing methods.

## Results

### Butt-seq is an efficient 3′ nascent RNA end-labeling technique

Nascent RNA contains a heterogeneous mixture of molecules in varying states of synthesis. They include short transcripts associated with paused RNA polymerase, partially synthesized RNAs, and completed transcripts awaiting cleavage and polyadenylation (Wissink et al. 2019). Butt-seq was inspired by other 3′ end-labeling techniques and captures the entire breadth of all nascent transcripts. However, the data presented focus on transcripts from protein-coding genes.

**Figure 1.**
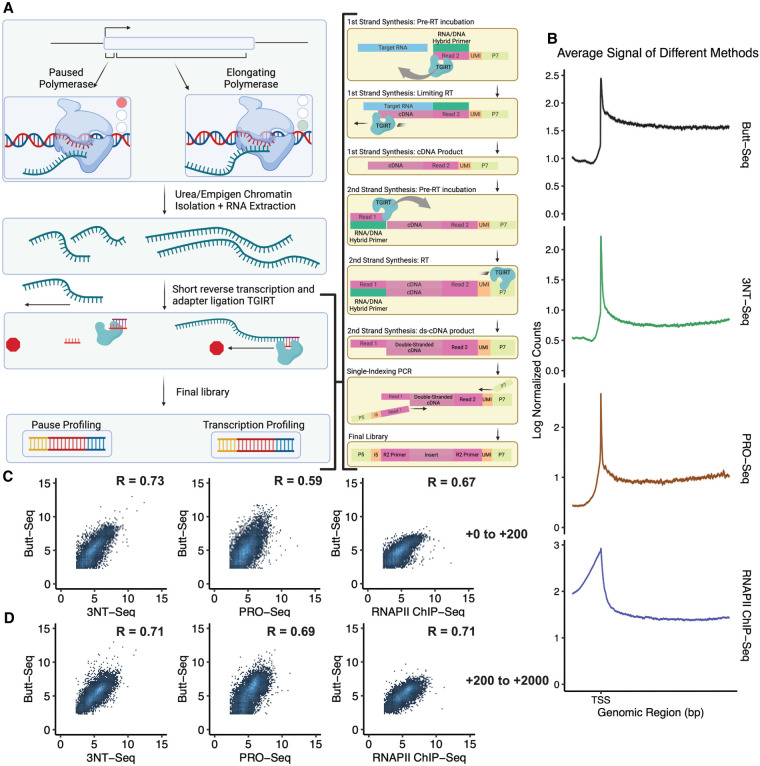
Butt-seq measures transcription and is comparable with other transcription analysis methods. (*A*) Butt-seq method overview. The figure was created with BioRender. (*B*) Scaled metagene plots of signal distribution in plus-strand genes >5 kb in length (*N* = 6284) across Butt-seq, 3NT-seq, PRO-seq, and RNAPII ChIP-seq. On the *X*-axis, the transcription start site (TSS) is marked, and the plot extends 1 kb into the gene body and 200 bp upstream. The *Y*-axis represents the log_2_ transformed normalized signal in each method. The shaded area corresponds to the 95% confidence interval. (*C*) Log–log plot of normalized counts from Butt-seq compared with 3NT-seq, PRO-seq, and RNAPII ChIP-seq genome-wide (*N* = 10,744). Signal was quantified from the region 200 nt downstream from the TSS genome-wide. The *Y*-axis represents the log_2_ transformed Butt-seq counts, while the *X*-axis represents the log_2_ transformed 3NT-seq (*left*), PRO-seq (*middle*), and RNAPII ChIP-seq (*right*) counts. Correlations were calculated using Spearman's rank correlation coefficient. (*D*) Log–log plot of normalized counts from Butt-seq compared with 3NT-seq, PRO-seq, and RNAPII ChIP-seq genome-wide (*N* = 10,744). Signal was quantified from the region +200 nt to +2000 nt downstream from the TSS. The *Y*-axis represents the log_2_ transformed Butt-seq counts, while the *X*-axis represents the log_2_ transformed 3NT-seq (*left*), PRO-seq (*middle*), and RNAPII ChIP-seq (*right*) counts. Correlations were calculated using Spearman's rank correlation coefficient.

The protocol first isolates chromatin with 0.5 M urea and 1.5% empigen as previously described (Rodriguez et al. 2013; Schlackow et al. 2017; Sousa-Luís et al. 2021). The resulting chromatin-associated RNA undergoes simultaneous adapter ligation and reverse transcription using the TGIRT reverse transcriptase and an adapter containing the read 2 sequence. There is also an 8-bp UMI in place of the usual i7 index as well as the P7 sequence. A short reverse transcription step is conducted with a low concentration of dNTPs; this is to limit the length of the cDNA and minimize length-dependent biases when sequencing. The final insert should be no longer than 350 nt in length. Furthermore, pre-RT incubation is done with a low concentration of salt, while dNTPs are added along with a higher concentration of salt; this is done to maximize the efficiency of the initial template switch but to minimize the amount of secondary template switches as well as slow down reverse transcription. Second strand synthesis and read 1 adaptor ligation are conducted through a second TGIRT reaction, and the final adapter-tagged cDNA is subjected to PCR with standard i5 indexing primer, gel extraction, and standard Illumina sequencing. The resulting library should reflect all chromatin-associated RNA, most notably a high-resolution view of polymerase pausing and transcriptional elongation (Fig. 1A).

### Butt-seq results are comparable with those from other established methods

A major use of 3′ end-labeling techniques is the identification and characterization of promoter-proximal pausing, represented by regions of distinctly high signal adjacent to the transcription start site (Mayer et al. 2017). Such pausing represents a distinct regulatory step: RNAPII transcribes a short stretch of RNA before pausing and awaiting further signaling before either disengaging or proceeding into processive elongation. To validate Butt-seq for pausing analysis, we examined the average signal distribution 200 nt downstream from the TSS genome-wide in *Drosophila* S2 cells and compared it with the signal from published data in these cells generated by other nascent RNA techniques: 3NT-seq, PRO-seq, and RNAPII ChIP-seq (Fig. 1B). All four techniques feature a sharp peak directly downstream from the TSS, likely driven by the high prevalence of promoter-proximal pausing across the genome, followed by signal reflecting transcripts undergoing processive elongation.

We quantified these regions and performed a log–log comparison of Butt-seq against the three published techniques. Butt-seq has the highest correlation to the most chemically analogous technique, 3NT-seq (Spearman correlation = 0.73), but also correlates well with RNAPII ChIP-seq (Spearman's correlation = 0.67) and PRO-seq (Spearman's correlation = 0.59). These relationships apply similarly to the gene body, where Butt-seq also correlates well with 3NT-seq (Spearman's correlation = 0.71), PRO-seq (Spearman's correlation = 0.69), and RNAPII ChIP-seq (Spearman's correlation = 0.71). These comparisons indicate that Butt-seq faithfully assays nascent RNA.

One point of concern in 3′ end-labeling techniques is the presence of small RNA and/or rRNA contaminants, which can drive up experimental costs by increasing the amount of required sequencing depth. 3NT-seq incorporates a terminator exonuclease step to degrade uncapped RNA species to reduce the number of undesirable analytes (Weber et al. 2014); Butt-seq forgoes this step. Nonetheless, the extent of small RNA and rRNA contamination is similar between the two techniques (Supplemental Fig. S1A,B). As expected, PRO-seq exhibits virtually no small RNA contamination and ∼15% less rRNA contamination. In any case, Butt-seq produces data of a quality comparable with those of existing techniques in its class. As with other 3′ end-labeling techniques, small RNAs and other contaminants are removed computationally prior to downstream analyses.

### Butt-seq is reproducible down to 10,000 cells

**Figure 2.**
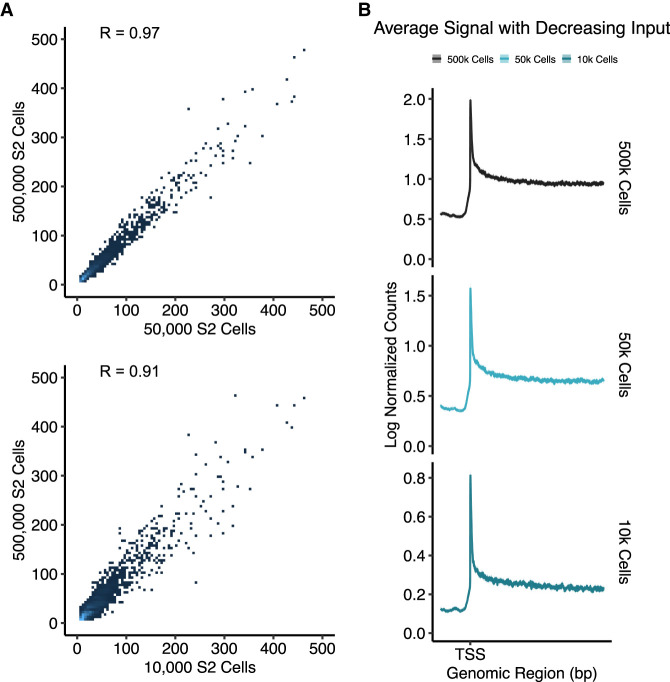
Butt-seq signal is reproducible down to 10,000 cells. (*A*) Comparison of signal from Butt-seq data generated from 500,000, 50,000, and 10,000 cells. Signal was quantified from 200 nt downstream from the TSS genome-wide. The *Y*-axis represents the log_2_ transformed counts from 500,000 cells, while the *X*-axis represents the log_2_ transformed counts from 50,000 (*top*) and 10,000 (*bottom*) cells. (*B*) Metagene plots of signal distribution in plus-strand genes >5 kb in length (*N* = 6284) from 500,000, 50,000, and 10,000 cells. On the *X*-axis, the transcription start site (TSS) is marked, and the plot extends 1 kb into the gene body and 200 bp upstream. The *Y*-axis represents the log_2_ transformed normalized signal in each method. Data were normalized to the depicted region The shaded area corresponds to the 95% confidence interval.

We next assayed the minimum input requirement for Butt-seq. Our eventual goal is to assay primary tissues or cells from which material may be limited. The Butt-seq signal 200 nt downstream from the TSS correlated well between 500,000 and 50,000 cells (Spearman's correlation = 0.97), and even between 500,000 and 10,000 cells (Spearman's correlation = 0.91) (Fig. 2A). There is a similar signal distribution pattern: All feature a sharp peak at the TSS indicative of promoter-proximal pausing (Fig. 2B). The high correlation coefficients indicate that Butt-seq is not only highly reproducible but also consistent with 10,000 cells or less.

### KL-1 causes pause sites to recede toward the TSS

We next used Butt-seq to characterize the effect of the AFF4/lilliputian (*lilli*) inhibitor KL-1 on transcription in S2 cells. KL-1 disrupts the formation of the superelongation complex (SEC) by inhibiting *lilli* activity (Liang et al. 2018). It thereby impedes the release of paused polymerase into productive elongation and attenuates the rate of elongation. As KL-1 has previously been shown to result in an increased RNAPII ChIP-seq signal at the TSS, we compared Butt-seq signal with published RNAPII ChIP-seq data (Liang et al. 2018).

**Figure 3.**
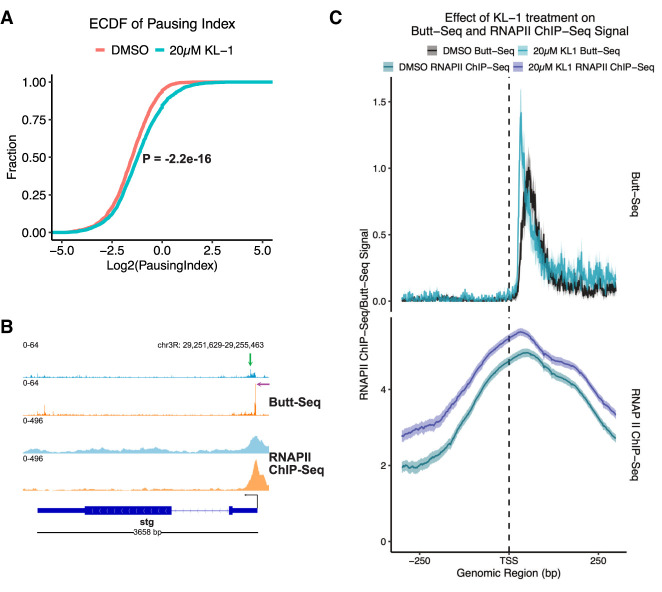
Butt-seq recapitulates pausing dynamics seen in RNAPII ChIP-seq upon KL-1 treatment. (*A*) ECDF of the pausing index in Butt-seq in S2 cells treated with DMSO or 20 µM KL-1. The pausing region is defined as the region from the TSS to the highest pause site as determined by pause detection algorithm (PDA), and the gene body region is defined as 1000 nt downstream from each pause site. The *Y*-axis depicts the cumulative fraction of genes, while the *X*-axis contains the log_2_ transformed pausing index. (*B*) Representative gene demonstrating the effect of 20 µM KL-1 treatment on signal distribution in Butt-seq and RNAPII ChIP-seq. (Green arrow) Untreated pause site, (purple arrow) KL-1-induced pause site. (*C*) Metagene plots of log_2_-normalized Butt-seq and RNAPII ChIP-seq counts, centered around RNAPII ChIP-seq peaks called from DMSO-treated S2 cells that overlap with Butt-seq single-nucleotide pause peaks. Only plus-strand genes are depicted (*N* = 219). The shaded region represents the 95% confidence interval.

To assay the genome-wide pausing index of 20 µM KL-1-treated S2 cells compared with vehicle (DMSO)-treated cells, we calculated the signal ratio between promoter-adjacent reads and gene body reads (Fig. 3A). Pausing index is usually calculated by dividing the signal in a fixed TSS-adjacent region by the signal in the gene body. The TSS-adjacent region is typically defined as the −50 region to the +300 region, although this can vary from study to study (Liang et al. 2018). While this definition has been used to great effect in ChIP-seq studies, single-nucleotide-resolution techniques enable more precision. We used a novel peak-calling algorithm developed for single-nucleotide techniques, the pause detection algorithm (PDA), to detect pauses in the first 200 bases downstream from the TSS (Gajos et al. 2021). We then filtered pauses to find the highest signal, so that each gene only had a single pause site. We then analyzed pause sites detected by PDA for motifs and identified an enrichment of G/A and T/C residues at the −1 and +1 nucleotide, respectively, similar to previously reported motifs (Supplemental Fig. S2A). The enriched motifs further indicate that these are proper pause sites, and we therefore defined the pausing region as extending from the TSS to the pause site, and the gene body as the region 2 kb downstream from the pause site. By measuring the pausing index on an empirical cumulative distribution function (ECDF) plot, there was a significant increase in pausing index upon KL-1 treatment (Kolmogorov–Smirnov, *D* = 0.11815, *P* < 2.2 × 10^−16^), as had been previously reported in RNAPII ChIP-seq data (Fig. 3A; Liang et al. 2018). We also calculated the pausing index using the standard method. It revealed the same degree of change, albeit at a lower magnitude (Kolmogorov–Smirnov, *D* = 0.0424, *P* < 0.001) (Supplemental Fig. S2A).

To analyze transcriptional dynamics at single-nucleotide resolution, each Butt-seq read was truncated to the first base sequenced by read 2; this should reflect the last nascent RNA base synthesized by RNAPII. Surprisingly, this single-nucleotide resolution indicated that KL-1 not only increased the magnitude of pausing as previously reported but also caused genome-wide pause sites to move upstream toward the TSS (a representative gene is shown in Fig. 3B; Liang et al. 2018). Reanalysis of published RNAPII ChIP-seq data and metagene profiling of this RNAPII ChIP-seq signal indicate that both conclusions are visible in these previous data (Fig. 3C). First, the Butt-seq signal peaks align with the RNAPII ChIP-seq peaks for both DMSO- and KL-1-treated conditions. Second, treatment with KL-1 causes the signal to move in the 5′ direction toward the TSS while also increasing in magnitude, suggestive of increased pausing in addition to altering the precise pause sites (Fig. 3C). This effect is most pronounced in Butt-seq, although it is also visible at lower resolution in the RNAPII ChIP-seq data.

### RNAPII positioning and KL-1 sensitivity correlate with subnucleosomal structures of the −1 nucleosome

**Figure 4.**
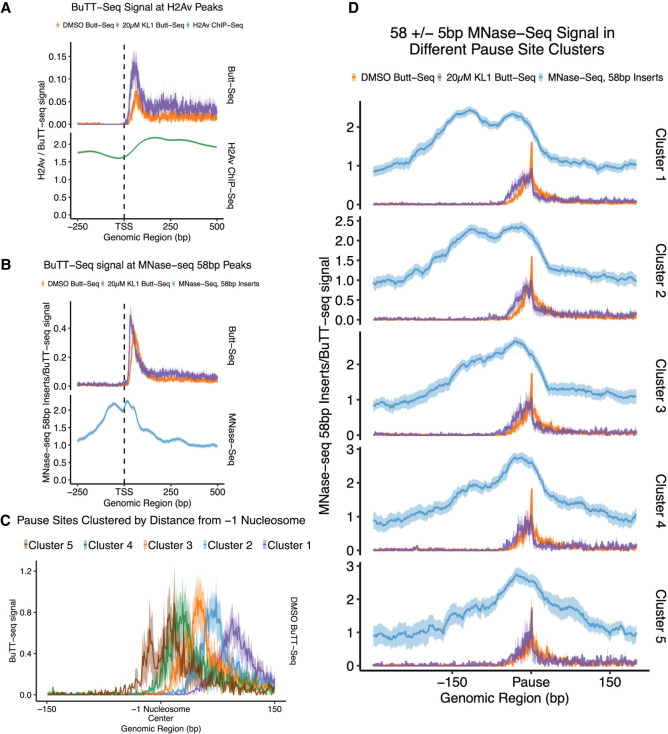
Pausing as assayed by Butt-seq is correlated with nucleosomal dynamics. (*A*) Metagene plots of log_2_ transformed Butt-seq counts compared with log_2_-normalized MNase-seq counts, restricted to 58-bp ± 5-nt fragments from MNase-seq. Plotted genes were derived from MNase-seq peaks that overlap with annotated TSS; plus-strand genes are shown (*N* = 831). The shaded area corresponds to the 95% confidence interval. (*B*) Metagene plot of log_2_ transformed H2Av ChIP-seq counts compared with log_2_ transformed Butt-seq counts from S2 cells treated with DMSO or 20 µM KL-1. Plotted genes were derived from H2Av ChIP-seq peaks that overlap with annotated TSS; plus-strand genes are shown (*N* = 1531). The shaded area corresponds to the 95% confidence interval. (*C*) Clustering of pause peaks based on distance from the −1 nucleosome center. Depicted are plus-strand genes. Cluster 1 contains pauses 80–120 bp downstream (*N* = 140), cluster 2 contains pauses 60–79 bp downstream (*N* = 95), cluster 3 contains pauses 40–59 bp downstream (*N* = 63), cluster 4 contains pauses 20–39 bp downstream (*N* = 45), and cluster 5 contains pauses 0–19 bp downstream (*N* = 35). The shaded area corresponds to the 95% confidence interval. (*D*) Metagene plot of log_2_-normalized 58-bp ± 5-bp MNase-seq counts against log_2_-normalized KL-1- and DMSO-treated Butt-seq counts across five clusters. The shaded area corresponds to the 95% confidence interval.

We leveraged the single-nucleotide sensitivity of Butt-seq to address the relationship between RNAPII pausing and subnucleosome structure. MNase-seq is used to measure nucleosome positioning, and selective sequencing and analysis of subnucleosomal (<147 bp) MNase-digested fragments can be used to map partially unwrapped subnucleosomal particles (Ramachandran et al. 2017). We therefore examined the relation of Butt-seq signal to previously published subnucleosomal MNase-seq data from S2 cells (Ramachandran et al. 2017). These fragments typically exhibit a bimodal distribution, representing protection from digestion by the two halves of a partially unwrapped histone dyad (visible in Fig. 4A, top). RNAPII proximity to the +1 nucleosome correlates with loss of the dyad distal to the TSS (Ramachandran et al. 2017). This observation was inferred to be a causal result of RNAPII transcribing through the nucleosome. However, many studies suggest the opposite: Even the subnucleosome particle is a transcriptional barrier capable of slowing and pausing RNAPII, which then can be overcome by RNAPII through a number of molecular strategies. In any case, we asked whether subnucleosomal dynamics correlate with KL-1 sensitivity.

We first measured the genome-wide distribution of the Butt-seq signal and MNase-seq signal restricted to 58-bp ± 5-bp fragments (Fig. 4A). A surprising number of MNase peaks with a bimodal distribution overlapped the TSS (*N* = 1684) (visible in Fig. 4A, top). The region overlapping the TSS is typically thought to be a nucleosome-free region, and the −1 nucleosome is defined as the region just preceding the TSS. However, this 58-bp pattern is consistent with protection by a subnucleosomal H3–H4 dyad, a distribution referred to as a −1 nucleosome (Ramachandran et al. 2017); we use this terminology here. Pausing was enriched in genes exhibiting this fragment distribution pattern and was also sensitive to KL-1 treatment (Fig. 4A, bottom). In contrast, pausing was depleted in genes enriched with the H2Av variant in the +1 nucleosome as previously shown (Fig. 4B; Weber et al. 2014). Surprisingly, however, H2Av-enriched genes were also sensitive to KL-1 treatment.

To further analyze these Butt-seq pause sites, we divided them into five clusters based on their distance downstream from the center of the −1 nucleosome (Fig. 4C). Cluster 1 contains pauses 80–120 bp downstream from the −1 nucleosome, cluster 2 contains pauses 60–79 bp downstream, cluster 3 contains pauses 40–59 bp downstream, cluster 4 contains pauses 20–39 bp downstream, and cluster 5 contains pauses 0–19 bp downstream. To examine the relationship between the pausing clusters, KL-1 sensitivity, and nucleosome occupancy, we plotted MNase-seq and KL-1-treated Butt-seq signals against the DMSO-treated signal in each cluster; this revealed two notable patterns (Fig. 4D).

First, the character of the nucleosome dyad changes in each cluster. In cluster 1, where the pause site is furthest from the −1 nucleosome, both halves of the nucleosomal dyad are present. In cluster 5, where the pause site is very close to the +1 nucleosome, the distal half of the nucleosome dyad is completely absent. Moving from cluster 1 to cluster 5, there is a gradual loss of the nucleosome dyad distal half (Fig. 4D, blue). Second, the effect of KL-1 treatment on the pause site appears to be impacted by the distance of the pause site to the nucleosome center; the further downstream the pause site, the greater the effect of KL-1.

### Butt-seq identifies differential pausing dynamics between S2 cells and *Drosophila* heads

**Figure 5.**
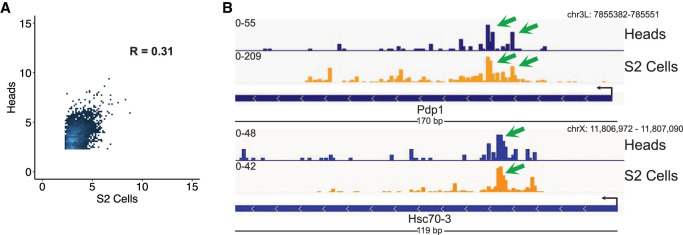
Despite low correlation in gene expression, S2 cells and heads share similar pausing features in coexpressed genes. (*A*) Comparison of counts from Butt-seq data generated from S2 cells or heads. Signal was quantified from 200 nt downstream from the TSS genome-wide. The *Y*-axis represents the log_2_ transformed counts from heads, while the *X*-axis represents the log_2_ transformed counts from S2 cells. (*B*) Representative genome browser view of genes exhibiting similar pause sites in heads and S2 cells. (Green arrow) Representative shared pause sites.

In contrast to the extensive amount of research conducted on tissue-specific gene expression programs, tissue-specific pausing has received rather little attention (Day et al. 2016). The extent to which an identically expressed set of genes may be differentially regulated by pausing is therefore poorly understood. One of the primary advantages of Butt-seq is that its more relaxed input material requirements enable a more facile profiling of primary tissues. To exploit this feature, Butt-seq was used to profile nascent RNA from a small number (*n* = 5) of Canton-S *Drosophila* heads and compared with the S2 cell profiles. Not surprisingly, these two very different tissues are poorly correlated (Spearman's correlation = 0.30) (Fig. 5A). However, many commonly expressed genes have similar single-nucleotide pausing signatures (Fig. 5B).

**Figure 6.**
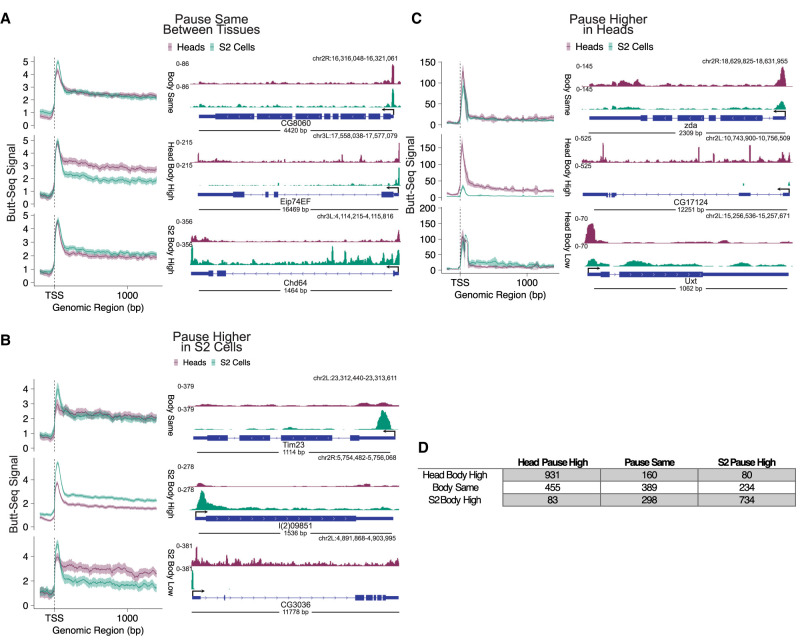
Differential analysis of Butt-seq in heads and S2 cells reveals a diverse range of transcriptional programs. Metagene analysis (*left*) and representative genes (*right*) reflecting different combinations of pause region and gene body signal between S2 cells and heads. (*A*) Pausing is the same between heads and S2 cells. (*B*) Pausing is higher in S2 cells. (*C*) Pausing is higher in heads. (*D*) Total number of genes occupying each pause/gene body combination.

We sought to explore the extent to which transcriptional programs might vary between the two tissues. For example, are there genes commonly expressed between the two tissues, but elongation in only one tissue is gated by a pause–release step? To further explore the variation in pausing and elongation dynamics between heads and S2 cells, we first called single-nucleotide peaks separately from S2 cells and heads, restricting the peak calling to within 200 nt of the TSS and filtering for the highest signal when a gene had multiple peaks. We then merged peaks between S2 cells and heads and compared signals in pausing regions and elongation regions of each gene: We defined the pause region as the region between the pause peak and the TSS, and the elongation region as a gene body region 1000 bp downstream from the pause peak. We then compared the relative signal between the pause region and the elongation region between S2 cells and heads (Fig. 6).

Pausing and elongation are concordant in 61% of genes; when pausing is higher in one tissue, elongation is also higher and vice versa (Fig. 6A [top], B,C [middle], D). These genes might simply manifest tissue differences in rates of transcriptional initiation. In most of the other genes (31%), there was a discordant relationship between pause and elongation regions: Heads and S2 cells exhibit the same signal in one parameter but a different signal in the other (Fig. 6B,C, top and bottom). Most notably, 8% of genes are discordant for both parameters; e.g., when pausing is higher in one tissue, elongation is lower in the other. These genes may therefore experience differential bottlenecking by pause release factors, suggesting the presence of different transcriptional programs regulating commonly expressed genes.

### Butt-seq identifies transcriptional dynamics in key circadian genes

We and others have previously measured the association of RNAPII with circadian clock genes in fly heads at different times of day (Abruzzi et al. 2011); for example, by ChIP-seq. Taken together with other assays (Taylor and Hardin 2008), there is strong evidence that RNAPII cycles on and off clock gene chromatin in a circadian manner and parallels cycling clock gene transcription. However, there is one striking exception: There are substantial levels of RNAPII stably associated with the *period* (*per*) gene promoter region at all times of day, even at times of day when there is little transcription (Taylor and Hardin 2008; Abruzzi et al. 2011). Will Butt-seq recapitulate or perhaps even extend these previous observations? To answer this question, we assayed fly head nuclei from entrained flies collected at six times of day, ZT2–ZT22 (ZT is time in a 12:12 LD cycle, with ZT0 indicating lights on and ZT12 indicating lights off).

**Figure 7.**
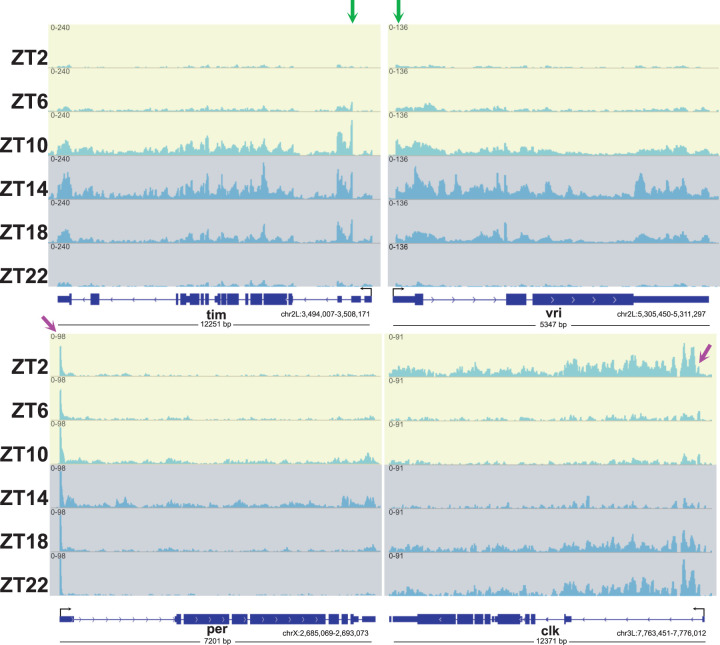
Butt-seq exhibits transcriptional cycling of core circadian genes. Genome browser view of six time points of core circadian genes in Butt-seq. (Green arrow) Cycling pause, (purple arrow) constant pause.

As anticipated, the clock gene body signal undergoes robust circadian cycling with peaks at ZT14 and troughs at ZT2 for the CLK/CYC direct target genes *per, vri*, and *tim*, and a peak at ZT2 and a trough at ZT14 for the clock gene *Clk*. (Fig. 6). Notably, the *per* Butt-seq data show a prominent and stable signal across peak and trough time points just downstream from the transcription start site (Fig. 7A, top left). This indicates that pause release might be under circadian regulation and contribute to *per* transcription. In contrast, the other two direct target genes *vri* and *tim* differ from *per.* Although *tim* and *vri* show notable pause sites near their TSSs (Fig. 7A, top right and bottom left), they oscillate with an amplitude similar to that of the gene body (Fig. 7A; data not shown). This suggests that the transcription of these two genes is primarily regulated through oscillating transcription initiation. In contrast, the *Clk* gene features a much smaller but temporally constant pause site like that of *per*, which implies that *Clk* may also be at least partially regulated through pause release.

**Figure 8.**
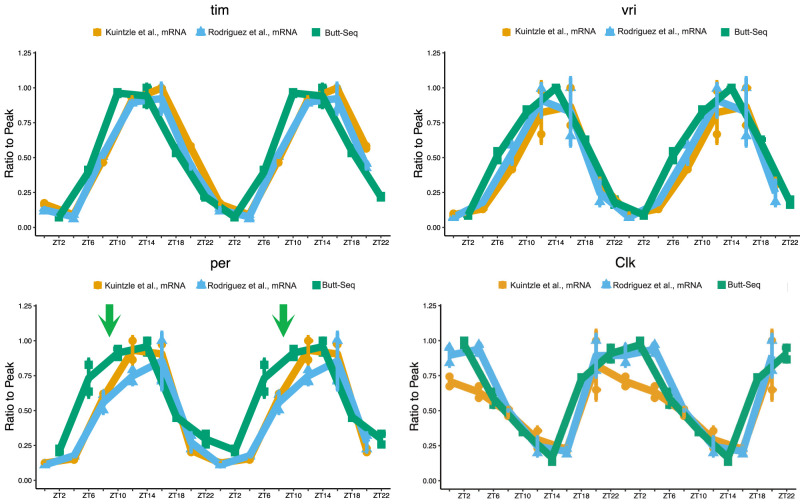
Butt-seq recapitulates known transcriptional features of the circadian clock. Butt-seq double-plotted against RNA-seq data from Rodriguez et al. (2013) and Kuintzle et al. (2017) at core circadian genes. Each time point was normalized to the peak time point in respective genes. (Green arrow) “Hump” of transcription in per gene.

### Butt-seq recapitulates circadian transcriptional oscillation details

Could Butt-seq be used to assess different transcription rates? Although a high Butt-seq signal in gene body regions may reflect a high level of RNA production, it may also reflect a high level of RNAPII occupancy due to a slow rate of transcriptional elongation. However, both NET-seq and RNAPII ChIP-seq signals have previously been used to approximate transcription levels of RNA synthesis because RNAPII occupancy often if not usually reflects RNA synthesis. To examine in quantitative detail this putative circadian transcriptional regulation, we calculated the Butt-seq signal within every gene at each of the six time points, and only exon signals were summed; i.e., intron signals were removed to avoid possible complications from nascent splicing. To help interpret the data, we turned to two published circadian mRNA-seq data sets from *Drosophila* heads: one generated by our laboratory almost 10 yr ago, and the other generated by another group 5 yr ago (Rodriguez et al. 2013; Kuintzle et al. 2017). For ease of illustration, we focused here on the four core clock direct CLK target genes presented above and double-plotted these two data sets along with our Butt-seq data (Fig. 8).

A first notable observation is that the two RNA-seq data sets are very similar; the *tim* and *vri* curves are indistinguishable, whereas only the peak values of the *Clk* profiles and, to a lesser extent, the *per* profiles are somewhat different (Fig. 7B, purple and blue). A second observation is that the Butt-seq data are virtually superimposable onto the RNA-seq data for three of the four genes. This suggests that transcriptional regulation accounts for almost all mRNA dynamics for these four core clock genes (Fig. 7B).

The only notable exception is *per*. The Butt-seq data indicate that the mid-day increase in transcription is phase-advanced relative to the RNA-seq data. Because the nighttime decreases are coincident, this makes for a broader Butt-seq plateau between ZT10 and ZT14 relative to the RNA-seq data (Fig. 7B, green arrows). Remarkably, this comparison is identical to what we reported in a comparison between a nuclear run-on assay of *per* transcription and an RNase protection assay of steady-state *per* mRNA (So and Rosbash 1997). Those assays were low-throughput, limiting the number of genes that could be examined. Nonetheless, *tim* was assessed in parallel and was more equivocal; i.e., it did not show such a striking distinction between transcription and mRNA levels (So and Rosbash 1997). This is similar to what we observed here between *tim* Butt-seq and RNA-seq data (Fig. 7B). The parallels between these two different assays done 25 yr apart indicate that the circadian regulation of *per* is quantitatively and perhaps even qualitatively unusual compared with the other core clock genes and most importantly support the assertion that the Butt-seq exon signal successfully assays transcription.

## Discussion

Butt-seq is a facile method for the analysis of nascent RNA and sequences the 3′ ends of chromatin-associated RNA. In most analyzed reads, the 3′ end is the base most recently synthesized by RNAPII. Although this same strategy is used by a wide range of methods, Butt-seq offers a much simpler workflow: It can produce libraries from purified nascent RNA in as few as 6 h and is reproducible down to 10,000 cells.

Although the choice of TGIRT for reverse transcription and adaptor ligation is the most notable difference from these previous methods, many of them use selection steps that Butt-seq does not; for example, phosphorylation state-specific immunoprecipitation of RNAPII. scRNA-seq and 3NT-seq also deplete uncapped RNA species with a cap-sensitive exonuclease. Although Butt-seq circumvents this step, it nevertheless correlates well with 3NT-seq (Fig. 1B,C), suggesting that cap selection may not have a large effect on signal in regions of interest. Relative to these earlier methods, Butt-seq bears the greatest resemblance to hNET-seq, which also does not incorporate a selection step. Despite the lack of a polymerase-bound or capped RNA selection in the Butt-seq protocol, it shows similar signals at protein-coding genes with a much simplified workflow and improved input requirements.

The capacity of Butt-seq to address transcriptional pausing in detail is highlighted in experiments demonstrating that the transcriptional elongation and SEC inhibitor KL-1 causes transcriptional pause sites to recede toward the TSS. The extent of this movement was relatively short, ∼10–80 bp, making the possibility that this change in signal is due to changes in TSS selection unlikely. Furthermore, we did not observe widespread TSS selection changes upon KL-1 treatment. This observation implies the existence of multiple pausing checkpoints downstream from the TSS, the closest of which is rapidly resolved by the recruitment of the SEC shortly after transcriptional initiation. The existence of multiple pausing checkpoints resembles the recently proposed “pausing zone,” a region of enhanced RNA polymerase pausing adjacent to the transcription start site (Fong et al. 2022). Notably, this study reported that a phosphorylation mutant of Spt5, Spt5 KOWx4–KOW5, exhibited increased signal density in regions of the pausing zone adjacent to the TSS, suggesting that this phosphorylation mutant impedes the ability of RNAPII to exit this early pausing zone. This pausing zone finding corroborates our observation of multiple pause sites and suggests that this mode of pause–release regulation may be conserved between insects and mammals. Unlike the study focused on the KOWx4–KOW5 mutant, however, we focused our analysis on the shifting of discrete major pause sites. In addition, close examination of the metagene plots suggests that KL-1 treatment causes increased signal at least 500 bp into the gene body, which hints that a pausing zone exists in our data as well.

These pause sites have an intriguing relationship to subnucleosomal fragments obtained through MNase-seq: Increased proximity of the pause site to the −1 nucleosome center is negatively correlated with sensitivity to KL-1 treatment and positively correlated with a loss of contact with the distal half of the nucleosomal dyad. These differences in nucleosomal contacts may be a consequence of RNAPII proximity as previously suggested (Ramachandran et al. 2017). However, the insensitivity to KL-1 in genes with pause sites directly adjacent to the center of the nucleosome suggests more complex regulation. Surprisingly, KL-1 appears to also cause transcriptional deceleration near the TSS in genes containing H2Av in the +1 nucleosome, which is depleted of promoter-proximal pausing in *Drosophila* (Weber et al. 2014). This suggests that recruitment of the SEC might occur concurrently with transcriptional initiation at H2Av-marked genes, which then bypasses promoter-proximal pausing.

A strength of Butt-seq should be its ability to interrogate samples where input material may be limited, like primary tissue. To this end, we assayed fly heads with Butt-seq at six different time points and focused on four clock genes. *per* features a very prominent stable pause signal, as previously observed. Future experiments should indicate whether the rhythmic transcription of *per* is regulated at least in part through pause release. In this context, there is evidence that *per* mRNA is more stable early in its accumulation cycle (So and Rosbash 1997). This is when pause release should be prominent, before the large RNAPII increase in the elongating region. Perhaps there is a difference in messenger ribonucleoprotein (mRNP) composition between transcripts due to pause release compared with those that accumulate later due to increased transcriptional initiation.

In this context, we have previously explored the relative contributions of transcriptional versus post-transcriptional regulation to circadian mRNA oscillations. Given that measuring Butt-seq signal along the gene body represents an uncommon approach toward examining transcriptional dynamics (essentially, the quantification of RNAPII density), we revisited this question in a simple way by examining the relationship between Butt-seq signal and mRNA-seq signal. Interestingly, the amplitude, period, and phase of the Butt-seq signal and the mRNA-seq signal were remarkably similar, with the Butt-seq phase leading mRNA-seq by no more than ∼1 h. A genome-wide analysis of oscillating transcription suggests that aspects of this relationship may be general (data not shown); however, the complexity of this analysis puts it outside the scope of this methods report. We therefore chose to focus on the core circadian clock components whose transcriptional regulation have been well characterized over decades and multiple biochemical approaches. In one previous study using nuclear run-on and RNA-radiolabeling methods, we reported a “hump” of *per* transcription that precedes the peak of *per* mRNA; comparing the two curves suggested interesting *per* post-transcriptional regulation in addition to its more expected transcriptional regulation (So and Rosbash 1997). The same “hump” is visible in Butt-seq now 25 yr later (Fig. 6B). Although further exploration is beyond the scope of this study, these observations as well as the paused polymerase peak in the *per* promoter region (Fig. 6A; Taylor and Hardin 2008; Abruzzi et al. 2011) suggest that *per* is subject to unusual modes of regulation relative to other CLK-regulated core circadian genes.

The extent of tissue-specific transcriptional pausing and pause release has received relatively little attention. The relationship between pausing and elongation was nonlinear in differentially expressed genes between heads and S2 cells; in other words, differences in pausing signal were frequently not accompanied by a concordant change in gene body signal. This finding echoes a previous study analyzing pausing through RNAPII ChIP-seq in different mammalian tissues (Day et al. 2016). One possibility is that pausing enables a second layer of regulation, wherein the combinatorial recruitment of initiation and pause release factors enables tissue-specific gene expression (Adelman and Lis 2012). This discordant relationship implies that pause release may be a rate-limiting step in the expression of many genes including *per*, and that interesting tissue-specific factors may be required to enable pause–release and the transition from pausing to processive elongation. In any case, we suggest that Butt-seq will provide future insights into transcriptional regulation from many other areas of investigation well beyond flies and circadian biology.

## Materials and methods

### S2 cell culture and KL-1 treatment

S2 cells were obtained from ATCC (CRL-1963) and cultured in Schneider's medium supplemented with 10% FetalGro (rmbio FGX-BBT) and 1% penicillin/streptomycin (Thermo Fisher 15140122). For KL-1 treatment, S2 cells were plated in six-well plates at a density of 1 × 10^6^ cells per well and left for 1 d. KL-1 was added to a final concentration of 20 µM or an equal volume of DMSO was added to the cells and treated for 6 h. Cells were harvested after 6 h.

### Nascent RNA isolation in S2 cells

Nascent RNA isolation was adapted from Khodor et al. (2012). S2 cells grown in a T25 flask were harvested by scraping, pelleted in a centrifuge at 900*g* for 5 min, and washed once with 10 mL of 1× cold PBS. Unless otherwise specified, 5 × 10^5^ cells were used. Washed cells were resuspended in 500 µL of cell lysis buffer (10 mM Tris at pH 7.5, 2 mM MgCl_2_, 10 mM KCl, 0.6 mM spermidine, 0.2 mM spermine, 3 mM TCEP, 0.03% Tween-20, 0.1% BSA) and transferred into 2 mL of dounce homogenizers. Cells were homogenized with 10 strokes with pestle A and 15 strokes with pestle B. Homogenized cells were passed through a 10-µm filter (Sysmex 04-0042-2314) and centrifuged at 1000*g* for 5 min. Supernatant was removed, and nuclei were resuspended in 100 µL of nuclear lysis buffer (10 mM HEPES-KOH at pH 7.6, 100 mM KCl, 0.1 mM EDTA, 10% glycerol, 0.15 mM spermine, 0.5 mM spermidine, 0.1 mM NaF, 0.1 mM Na_3_VO_4_, 0.1 mM ZnCl_2_, 1 mM TCEP, 0.1 U/µL SUPERaseIn, 1× cOmplete protease inhibitor), placed on a Thermomix set to 4°C, and shaken at 1400 rpm. One-hundred microliters of NUN buffer (25 mM HEPES-KOH at pH 7.6, 300 mM NaCl, 1 M urea, 1% NP-40, 1 mM TCEP, 3% empigen, 0.1 U/µL SUPERaseIn, 1× cOmplete protease inhibitor) was added dropwise while shaking. Tubes were capped and left shaking for 10 min at 4°C. Chromatin was pelleted in the centrifuge at 21,000*g* for 10 min, and the supernatant was discarded. The resulting chromatin pellet was resuspended in 500 µL of TRIzol, incubated for 10 min at 60°C, and then transferred to a phase lock tube. RNA was then isolated with chloroform according to standard procedure and resuspended in a 10-µL Turbo DNase reaction containing 1× Turbo DNase buffer and 1.5 µL of Turbo DNase, and DNase treatment was done for 30 min according to protocol. Two microliters of DNase-treated RNA was used for Butt-seq.

### Nascent RNA isolation in fly heads

Flies were frozen on dry ice. Five heads were removed on dry ice and transferred into a 2-mL dounce homogenizer. Five-hundred microliters of nuclear lysis buffer was added to each homogenizer, and heads were dounced for 10 strokes with pestle A and 20 strokes with pestle B. The resulting homogenate was first filtered through a 20-µm filter and then a 20-µm filter. Nuclei were spun down at 1000*g* for 5 min and washed once with 500 µL of nuclear lysis buffer. The resulting nuclei were subjected to chromatin isolation as with S2 cells.

### Butt-seq library preparation

Butt-seq library preparation consisted of two consecutive TGIRT protocols for first and second strand synthesis. For a detailed protocol, along with notes and rationale, please see the Supplemental Protocol. The first strand synthesis primer contained the read 2 sequencing primer, UMI sequence, and P7 sequence. The second strand synthesis primer contained only the read 1 sequencing primer. An i5 barcode and the P5 sequence were added through PCR (Fig. 1A).

RNA/DNA hybrid primer assembly was similar to the commercial protocol but with concentrations modified to reduce the volume of primer required in the reaction. Please see the Supplemental Primers for a complete table of primers used. To prepare SCR2R primer, 10 µL of 10 µM R2 RNA (rArArGrArUrCrGrGrArArGrArGrCrArCrArCrGrUrCrUrGrArArCrUrCrCrArGrUrCrArC/3SpC3/) was mixed with 10 µL of 10 µM SCR2 DNA (CAAGCAGAAGACGGCATACGAGATNNNNNNNNGTGACTGGAGTTCAGACGTGTGCTCTTCCGATCTTN, where N is a hand-mixed equimolar ratio of A/T/C/G), along with 5 µL of 10× annealing buffer (10 mM Tris-HCl at pH 7.5, 10 mM EDTA, 10 mM TCEP at pH 7.5) and 25 µL of H_2_O. SCR2R primer was ordered as PAGE-purified, while SCR2 DNA was ordered as a PAGE-purified Ultramer from IDT. Primers were added to a preheated 88°C thermal cycler, incubated for 2 min, then cooled to 10°C at a rate of 0.1°C/sec, and held at 10°C.

To prepare MER1R primer, 10 µL of 10 µM R1ME RNA (rCrUrGrUrCrUrCrUrUrArUrArCrArCrArUrCrUrGrArCrGrCrUrGrC/3SpC3/) was mixed with 10 µL of 10 µM R1ME DNA (GCAGCGTCAGATGTGTATAAGAGACAGN, where N is a hand-mixed equimolar ratio of A/T/C/G), along with 5 µL of 10× annealing buffer (10 mM Tris-HCl at pH 7.5, 10 mM EDTA, 10 mM TCEP at pH 7.5) and 25 µL of H_2_O (Rhee and Burke 2004). Both primers were PAGE-purified from IDT. Primers were added to a preheated 88°C thermal cycler, incubated for 2 min, then cooled to 10°C at a rate of 0.1°C/sec, and held at 10°C.

All primers were aliquoted into single-used aliquots and stored at −80°C for up to 6 mo.

First strand master mixes (FS-MMs) were prepared with 0.5 µL of 10× ButtRT buffer (100 mM HEPES at pH 8, 500 mM NaCl, 50 mM MgCl_2_, 10 mM TCEP), 0.2 µL of SCR2R, 0.2 µL of TGIRT, and 1.1 µL of 50% PEG3350. FS-MMs were prepared for at least four reactions at a time to minimize pipetting errors. To ensure the reactions were well mixed, reactions were stirred 10 times with a pipette tip and flicked and spun down twice. Two microliters of FS-MMs was added to 2 µL of RNA in a PCR strip tube and flicked and spun down twice to mix. RNA inputs between 0.5 pg and 25 ng have been tested, but when working with <500,000 cells, we did not quantify RNA prior to assembling the reaction. Assembled reactions were incubated for 30 min on ice.

While incubating, 1 µL of 5 mM dNTPs was mixed with 30 µL of 2 M NaCl. After 30 min, 1 µL of dNTP/NaCl mixture was added to TGIRT reactions and placed on the thermal cycler with the following program: 1 min at 25°C, ramping to 60°C at 1°C/sec, 10 sec at 60°C, and then held at 4°C. Reactions were moved onto ice, and 2 µL of exonuclease III, 2 µL of H_2_O, and 1 µL of NEBuffer 1 were added and flicked to mix. Reactions were incubated for 8 min at 37°C and then moved onto ice. Exonuclease III treatment partially digested unused primers. This step is unnecessary with sufficient input, but with low-input samples, excess primers may be overamplified and contaminate the final library.

To eliminate RNA from downstream reactions and disassociate TGIRT, 3 µL of 1 M NaOH was added to each sample and heated for 5 min at 95°C. After cooling to room temperature, 3 µL of 1 M HCl was added to each sample to neutralize the reaction. Twenty-four microliters of 95% EtOH and 24 µL of Ampure XP beads were added to each sample, flicked to mix, and left to incubate for 10 min at room temperature (Fishman and Lamm 2019). Beads were washed twice with 200 µL of 80% EtOH and resuspended in 4.2 µL of H_2_O, and 4 µL of supernatant was moved to fresh PCR tubes.

Second strand synthesis master mixes (SS-MMs) were prepared with 1 µL of ButtRT buffer, 0.3 µL of MER1R, 0.3 µL of TGIRT, 2 µL of 50% PEG3350, and 0.4 µL of H_2_O. SS-MMs were mixed as described earlier. Four microliters of SS-MMs was added to cDNA samples and mixed as described earlier, and assembled reactions were left to incubate for 30 min on ice.

While incubating, 1 µL of 20 mM dNTPs was mixed with 10 µL of 2 M NaCl. After 30 min, 2 µL of dNTP/NaCl mixture was added to TGIRT and placed on the thermal cycler with the following program: 1 min at 25°C, ramping to 60°C at 1°C/sec, 30 sec at 60°C, and then held at 4°C. Reactions were moved onto ice, and 1 µL of 0.2% SDS was added to each reaction to disassociate TGIRT. Reactions were incubated at 55°C and then moved to room temperature. Thirty microliters of Ampure XP beads was added to each reaction, incubated for 5 min at room temperature, washed twice, and then eluted in 9 µL of H_2_O.

dsDNA was added to PCR reactions containing 25 µL of 2× NEBNext Ultra II Q5 master mix, 2.5 µL of P7 primer (CAAGCAGAAGACGGCATACGAG), 2.5 µL of barcoded Ad1 primer (AATGATACGGCGACCACCGAGATCTACAC < barcode > TCGTCGGCAGCGTCAGATGTGTAT), and 11 µL of H_2_O. The assembled PCR reaction was subjected to the following PCR program: 3 min at 72°C, 30 sec at 98°C, nine to 15 cycles of 15 sec at 98°C, 30 sec at 65°C, a final extension of 2 min at 72°C, and held at 10°C.

PCR reactions were cleaned with 65 µL of Ampure XP beads following the standard protocol and resuspended in 10 µL of 1× purple loading dye. Samples were loaded onto an 8% TBE gel alongside 1 µL of TriDye ultralow-range DNA ladder mixed with 9 µL of 1× purple loading dye and run at 180 V for 45 min. Gels were poststained with 1× SYBR Gold for 5 min, and the smear above 150 nt was excised into a 0.5-mL tube. The maximum fragment size produced in this protocol should be ∼700 nt. For an example gel, please see the Supplemental Protocol.

The gel extraction consisted of a “crush and soak” protocol, slightly adapted (Zubradt et al. 2017). Using a 21-gauge needle, a hole was poked in the bottom of 0.5-mL tubes, which were nested inside a 2.0-mL tube and centrifuged for 21,000*g* for 3 min. If any gel fragments remained in the 0.5-mL tube, a second hole was poked and the tube was centrifuged again. Six-hundred microliters of 300 mM NaCl was added to each tube, and tubes were incubated for 15 min at 75°C or overnight at 4°C, preferably with agitation or shaking. Supernatant and gel fragments were transferred into a Costar Spin-X centrifuge tube filter with a wide-bore 1000-µL pipette tip—or a 1000-µL pipette tip with the tip cut off—and spun at 21,000*g* for 2 min. Filters were discarded, and 600 µL of isopropanol and 0.7 µL of GlycoBlue were added to each tube and left to incubate for at least 15 min at room temperature or for up to overnight at −20°C (Li et al. 2020). Samples were spun at 21,000*g* for 30 min, washed twice with 80% EtOH, and resuspended in 7–10 µL of MilliQ H_2_O. Two microliters was used for analysis on a Tapestation 4200 D1000 tape and quantified using peak quantification centered around 170 nt. Samples were sequenced on a NextSeq 500 with at least 8-bp dual-index reads. Using *Drosophil*a, we sequenced to a depth of at least 20 million paired-end reads.

### Butt-seq data processing

A snakemake file for analyzing Butt-seq is available at https://github.com/albertdyu/BuTTSeq, which includes all custom scripts mentioned. Prior to demultiplexing, RunInfo.xml was altered so that read 2 is not counted as an indexed read, and samples were demultiplexed using the i5 index. This will output three files: Read 1 corresponds to read 1, read 2 corresponds to the UMI, and read 3 corresponds to read 2. UMIs were extracted from read 2 and appended to reads 1 and 3 individually using umi_tools extract with the following command: extract -I READ2_UMI –extract-method = regex –bc-pattern = –read2-in = READ1_OR_3 –stdout = umiextracted_reads/filler.fq.gz–read2-out = OUTPUT.fq.gz (Smith et al. 2017). This will also produce a filler file, which can be deleted. Reads were trimmed using fastp and the following settings: -trim_poly_g –trim_poly_x -F 1 –adapter_sequence AAGATCGGAAGAGC –adapter_sequence_r2 CTGTCTCTTATA (Chen et al. 2018). Reads were subjected to two-pass mapping with STAR to dm6 using the following settings: –alignMatesGapMax 100,000 –outSAMstrandField intronMotif –outFilterMismatchNoverLmax 0.05 –outFilterMultimapNmax 1 –outSJfilterReads Unique. Aligned reads were converted to bam files with SAMtools, and deduplicated using umi_tools dedup (Li et al. 2009). Small RNAs and exon 3′ ends were computationally removed using SAMtools and a bed file containing small RNAs and exon 3′ ends extracted from RefSeq gene annotations, and soft clipping was removed prior to single-nucleotide read conversion using a custom script modified from ngsutils (Breese and Liu 2013).

### Contaminant analysis

For small and noncoding RNA analysis, coordinates for snoRNAs, snRNAs, and scaRNAs were extracted from RefSeq annotation files and used to generate a reference file for featurecounts. Reads mapped to small and noncoding RNAs were quantified using featurecounts, and percentages of reads mapping were reported.

For rRNA analysis, coordinates for rRNA genes were extracted from RefSeq annotation files and used to extract fasta sequences from the dm6 genome assembly. Extracted fasta files were used to build a bowtie2 index, and reads were mapped using bowtie2 with the following settings: –very-fast-local –phred33 –no-mixed –no-discordant –dovetail -I 10 -X 700. Percentages of mapped reads were reported.

### PRO-seq and 3NT-seq data processing

Fastq files were obtained from the SRA using fasterq-dump and aligned to dm6 using STAR with the following settings: –alignMatesGapMax 50,000 –outFilterMismatchNoverLmax 0.06 –outFilterMatchNmin 15 –outFilterMultimapNmax 1 –outSJfilterReads Unique. Aligned reads were converted to bam files with SAMtools (Li et al. 2009). Reads were truncated to the first nucleotide of read 2 using a custom script, get_SNR_bam.py, slightly adapted to fit this pipeline (Nojima et al. 2015).

### ChIP-seq data processing and peak calling

Fastq files were retrieved from the SRA using fasterq-dump and aligned to dm6 with bwa mem. Peaks were called using MACS2 using the following parameters: –nomodel –extsize 200 -q 0.01.

### Butt-seq pause analysis

Reads were truncated to the first nucleotide of read 2 using a custom script and converted into stranded bedgraph files using Deeptools (Ramírez et al. 2014; Nojima et al. 2016). Bedgraph files were restricted to the first 200 nt downstream from each TSS genome-wide, with overlapping genes being merged together.

### Pause site analysis

Single-nucleotide peaks were called using the pause detection algorithm (PDA) with a window size of 100, a minimum intensity of 5, 10,000 bootstraps, and a *P*-value threshold of 0.00001 (Gajos et al. 2021). Single-nucleotide peaks were used as a reference for quantifying single-nucleotide bam files using featureCounts, and multiple peaks in a single gene were filtered for the highest peak in R (Liao et al. 2014).

### Pausing index calculation

Rather than calculating pausing indices using fixed regions, pausing regions were calculated with reference to highest pause peak in each gene. Pause regions were defined as the region from the TSS to the highest pause, and elongation regions were defined as 1000 bp downstream from the highest pause. We also calculated pause index by measuring signal from −50 to +200 around the TSS and dividing it by signal across the gene body.

### Motif analysis

Single-nucleotide peaks were extended 10 nt in either direction with bedtools slop and used as a reference to retrieve fasta sequences from the dm6 reference genome using getfasta (Quinlan 2014). Motifs were generated from fasta sequences using the R package universalmotif.

### mNase-seq analysis

Fastq files were retrieved from the SRA using fasterq-dump and aligned to dm6 with bwa mem using default settings. Bam files were filtered by insert size using SAMtools. Nucleosome positions were called as previously described, except rather than filtering for the downstream nucleosome, entire alignments were first used for unbiased analysis. Following unbiased analysis, the −1 nucleosome was described as any nucleosome that overlapped an annotated TSS. Bedtools was used to determine nucleosome centers and to calculate the distance to the nearest Butt-seq pause peak (Quinlan 2014).

### H2Av ChIP-seq analysis

Peaks were called using MACS2 using the following parameters: –nomodel –extsize 200 -q 0.01 (Zhang et al. 2008).

### Data normalization

Across different tissues and techniques, the molecular composition of each sequencing library can vary dramatically. For example, PRO-seq contains no contaminating small RNA reads, while 3NT-seq and Butt-seq consist of ∼40% small RNA reads (Supplemental Fig. S1A). Normalization by library depth therefore would be inappropriate. Instead, we normalized data by coverage over regions of interest to control for differences in background using the median of ratios method used in DESeq2 (Love et al. 2014). For each analysis, bed files describing the features of interest were converted into saf files and used as a reference for counting using featureCounts (Liao et al. 2014). Raw count tables were imported into DESeq2, which we used to produce normalized count tables for correlation analyses and scaling factors. Scaling factors were used to produce normalized bigwig files for data visualization using deepTool's bamCoverage function (Ramírez et al. 2014). For metagene plots, signal over the depicted region was used to generate scaling factors.

### RNA-seq analysis

Fastq files were retrieved from the SRA using fasterq-dump and aligned to dm6 using STAR with the following settings: –alignMatesGapMax 50,000 –outFilterMismatchNoverLmax 0.06 –outFilterMatchNmin 15 –outFilterMultimapNmax 1 –outSJfilterReads Unique. Aligned reads were converted to BAM files with SAMtools (Li et al. 2009). Reads were counted using featurecounts using RefSeq genes as a reference (Liao et al. 2014). Normalized count files were generated using DESeq2 (Love et al. 2014). For each gene, reads across all time points were normalized to the highest time point and double-plotted using ggplot2 (Wickham 2016).

### Metagene profiles and gene plots

Mapped reads in each sequencing experiment were used to generate normalization factors with DESeq2 using counts at regions of interest as references, as described earlier (Love et al. 2014). To simplify visualization, only plus strand reads and genes were used. Coverage plots were scaled to specified regions using deepTools computematrix with a bin size of 1, and the output was used for further processing in R.

In R, outliers defined as the top and bottom 0.1% regions were removed, and a pseudocount of 1 was added to all positions and converted into log_2_, from which means and 95% confidence intervals were determined using the bootstrap method with 10,000 repetitions. Means and confidence intervals were plotted using ggplot2 (Wickham 2016). For some figure assembly, Plotgardener was used (Kramer et al. 2022).

### Data availability

Sequencing data have been deposited in the Gene Expression Omnibus (GEO) under the accession code GSE228595. Scripts for data processing and analysis are available at https://github.com/albertdyu/BuTTSeq.

## Supplementary Material

Supplemental Material

## References

[GAD350434YUC1] Abruzzi KC, Rodriguez J, Menet JS, Desrochers J, Zadina A, Luo W, Tkachev S, Rosbash M. 2011. *Drosophila* CLOCK target gene characterization: implications for circadian tissue-specific gene expression. Genes Dev 25: 2374–2386. 10.1101/gad.178079.111 22085964 PMC3222903

[GAD350434YUC2] Adelman K, Lis JT. 2012. Promoter-proximal pausing of RNA polymerase II: emerging roles in metazoans. Nat Rev Genet 13: 720–731. 10.1038/nrg3293 22986266 PMC3552498

[GAD350434YUC3] Begik O, Diensthuber G, Liu H, Delgado-Tejedor A, Kontur C, Niazi AM, Valen E, Giraldez AJ, Beaudoin JD, Mattick JS, 2023. Nano3P-seq: transcriptome-wide analysis of gene expression and tail dynamics using end-capture nanopore cDNA sequencing. Nat Methods 20: 75–85. 10.1038/s41592-022-01714-w 36536091 PMC9834059

[GAD350434YUC4] Breese MR, Liu Y. 2013. NGSUtils: a software suite for analyzing and manipulating next-generation sequencing datasets. Bioinformatics 29: 494–496. 10.1093/bioinformatics/bts731 23314324 PMC3570212

[GAD350434YUC5] Chen S, Zhou Y, Chen Y, Gu J. 2018. fastp: an ultra-fast all-in-one FASTQ preprocessor. Bioinformatics 34: i884–i890. 10.1093/bioinformatics/bty560 30423086 PMC6129281

[GAD350434YUC6] Churchman LS, Weissman JS. 2012. Native elongating transcript sequencing (NET-seq). Curr Protoc Mol Biol 98: 14.4.1–14.4.17. 10.1002/0471142727.mb0414s98 22470065

[GAD350434YUC7] Core LJ, Waterfall JJ, Lis JT. 2008. Nascent RNA sequencing reveals widespread pausing and divergent initiation at human promoters. Science 322: 1845–1848. 10.1126/science.1162228 19056941 PMC2833333

[GAD350434YUC8] Dahlberg O, Shilkova O, Tang M, Holmqvist PH, Mannervik M. 2015. P-TEFb, the super elongation complex and mediator regulate a subset of non-paused genes during early *Drosophila* embryo development. PLoS Genet 11: e1004971. 10.1371/journal.pgen.1004971 25679530 PMC4334199

[GAD350434YUC9] Day DS, Zhang B, Stevens SM, Ferrari F, Larschan EN, Park PJ, Pu WT. 2016. Comprehensive analysis of promoter-proximal RNA polymerase II pausing across mammalian cell types. Genome Biol 17: 120. 10.1186/s13059-016-0984-2 27259512 PMC4893286

[GAD350434YUC10] Fishman A, Lamm AT. 2019. QsRNA-seq: a protocol for generating libraries for high-throughput sequencing of small RNAs. Bio Protoc 9: e3179. 10.21769/BioProtoc.3179 PMC7854107 33654982

[GAD350434YUC11] Fong N, Sheridan RM, Ramachandran S, Bentley DL. 2022. The pausing zone and control of RNA polymerase II elongation by Spt5: implications for the pause-release model. Mol Cell 82: 3632–3645.e4. 10.1016/j.molcel.2022.09.001 36206739 PMC9555879

[GAD350434YUC12] Gajos M, Jasnovidova O, van Bömmel A, Freier S, Vingron M, Mayer A. 2021. Conserved DNA sequence features underlie pervasive RNA polymerase pausing. Nucleic Acids Res 49: 4402–4420. 10.1093/nar/gkab208 33788942 PMC8096220

[GAD350434YUC13] Hardin PE. 2005. The circadian timekeeping system of *Drosophila*. Curr Biol 15: R714–R722. 10.1016/j.cub.2005.08.019 16139204

[GAD350434YUC14] He Q, Johnston J, Zeitlinger J. 2015. ChIP-nexus enables improved detection of in vivo transcription factor binding footprints. Nat Biotechnol 33: 395–401. 10.1038/nbt.3121 25751057 PMC4390430

[GAD350434YUC15] Jonkers I, Lis JT. 2015. Getting up to speed with transcription elongation by RNA polymerase II. Nat Rev Mol Cell Biol 16: 167–177. 10.1038/nrm3953 25693130 PMC4782187

[GAD350434YUC16] Judd J, Wojenski LA, Wainman LM, Tippens ND, Rice EJ, Dziubek A, Villafano GJ, Wissink EM, Versluis P, Bagepalli L, 2020. A rapid, sensitive, scalable method for precision run-on sequencing (PRO-seq). bioRxiv 10.1101/2020.05.18.102277

[GAD350434YUC17] Khodor YL, Menet JS, Tolan M, Rosbash M. 2012. Cotranscriptional splicing efficiency differs dramatically between *Drosophila* and mouse. RNA 18: 2174–2186. 10.1261/rna.034090.112 23097425 PMC3504670

[GAD350434YUC18] Kramer NE, Davis ES, Wenger CD, Deoudes EM, Parker SM, Love MI, Phanstiel DH. 2022. Plotgardener: cultivating precise multi-panel figures in R. Bioinformatics 38: 2042–2045. 10.1093/bioinformatics/btac057 35134826 PMC8963281

[GAD350434YUC19] Kuintzle RC, Chow ES, Westby TN, Gvakharia BO, Giebultowicz JM, Hendrix DA. 2017. Circadian deep sequencing reveals stress-response genes that adopt robust rhythmic expression during aging. Nat Commun 8: 14529. 10.1038/ncomms14529 28221375 PMC5321795

[GAD350434YUC20] Li H, Handsaker B, Wysoker A, Fennell T, Ruan J, Homer N, Marth G, Abecasis G, Durbin R, Genome Project Data Processing S. 2009. The sequence alignment/map format and SAMtools. Bioinformatics 25: 2078–2079. 10.1093/bioinformatics/btp352 19505943 PMC2723002

[GAD350434YUC21] Li Y, Chen S, Liu N, Ma L, Wang T, Veedu RN, Li T, Zhang F, Zhou H, Cheng X, 2020. A systematic investigation of key factors of nucleic acid precipitation toward optimized DNA/RNA isolation. BioTechniques 68: 191–199. 10.2144/btn-2019-0109 32066262

[GAD350434YUC22] Liang K, Smith ER, Aoi Y, Stoltz KL, Katagi H, Woodfin AR, Rendleman EJ, Marshall SA, Murray DC, Wang L, 2018. Targeting processive transcription elongation via SEC disruption for MYC-induced cancer therapy. Cell 175: 766–779.e17. 10.1016/j.cell.2018.09.027 30340042 PMC6422358

[GAD350434YUC23] Liao Y, Smyth GK, Shi W. 2014. Featurecounts: an efficient general purpose program for assigning sequence reads to genomic features. Bioinformatics 30: 923–930. 10.1093/bioinformatics/btt656 24227677

[GAD350434YUC24] Love MI, Huber W, Anders S. 2014. Moderated estimation of fold change and dispersion for RNA-seq data with DESeq2. Genome Biol 15: 550. 10.1186/s13059-014-0550-8 25516281 PMC4302049

[GAD350434YUC25] Mahat DB, Kwak H, Booth GT, Jonkers IH, Danko CG, Patel RK, Waters CT, Munson K, Core LJ, Lis JT. 2016. Base-pair-resolution genome-wide mapping of active RNA polymerases using precision nuclear run-on (PRO-seq). Nat Protoc 11: 1455–1476. 10.1038/nprot.2016.086 27442863 PMC5502525

[GAD350434YUC26] Mayer A, di Iulio J, Maleri S, Eser U, Vierstra J, Reynolds A, Sandstrom R, Stamatoyannopoulos JA, Churchman LS. 2015. Native elongating transcript sequencing reveals human transcriptional activity at nucleotide resolution. Cell 161: 541–554. 10.1016/j.cell.2015.03.010 25910208 PMC4528962

[GAD350434YUC27] Mayer A, Landry HM, Churchman LS. 2017. Pause & go: from the discovery of RNA polymerase pausing to its functional implications. Curr Opin Cell Biol 46: 72–80. 10.1016/j.ceb.2017.03.002 28363125 PMC5505790

[GAD350434YUC28] Mohr S, Ghanem E, Smith W, Sheeter D, Qin Y, King O, Polioudakis D, Iyer VR, Hunicke-Smith S, Swamy S, 2013. Thermostable group II intron reverse transcriptase fusion proteins and their use in cDNA synthesis and next-generation RNA sequencing. RNA 19: 958–970. 10.1261/rna.039743.113 23697550 PMC3683930

[GAD350434YUC29] Nechaev S, Fargo DC, dos Santos G, Liu L, Gao Y, Adelman K. 2010. Global analysis of short RNAs reveals widespread promoter-proximal stalling and arrest of Pol II in *Drosophila*. Science 327: 335–338. 10.1126/science.1181421 20007866 PMC3435875

[GAD350434YUC30] Nojima T, Gomes T, Grosso ARF, Kimura H, Dye MJ, Dhir S, Carmo-Fonseca M, Proudfoot NJ. 2015. Mammalian NET-seq reveals genome-wide nascent transcription coupled to RNA processing. Cell 161: 526–540. 10.1016/j.cell.2015.03.027 25910207 PMC4410947

[GAD350434YUC31] Nojima T, Gomes T, Carmo-Fonseca M, Proudfoot NJ. 2016. Mammalian NET-seq analysis defines nascent RNA profiles and associated RNA processing genome-wide. Nat Protoc 11: 413–428. 10.1038/nprot.2016.012 26844429 PMC5152764

[GAD350434YUC32] Quinlan AR. 2014. BEDTools: the Swiss-army tool for genome feature analysis. Curr Protoc Bioinformatics 47: 11.12.1–11.12.34. 10.1002/0471250953.bi1112s47 PMC4213956 25199790

[GAD350434YUC33] Ramachandran S, Ahmad K, Henikoff S. 2017. Transcription and remodeling produce asymmetrically unwrapped nucleosomal intermediates. Mol Cell 68: 1038–1053.e4. 10.1016/j.molcel.2017.11.015 29225036 PMC6421108

[GAD350434YUC34] Ramírez F, Dündar F, Diehl S, Gruning BA, Manke T. 2014. Deeptools: a flexible platform for exploring deep-sequencing data. Nucleic Acids Res 42: W187–W191. 10.1093/nar/gku365 24799436 PMC4086134

[GAD350434YUC35] Rhee SS, Burke DH. 2004. Tris(2-carboxyethyl)phosphine stabilization of RNA: comparison with dithiothreitol for use with nucleic acid and thiophosphoryl chemistry. Anal Biochem 325: 137–143. 10.1016/j.ab.2003.10.019 14715294

[GAD350434YUC36] Rodriguez J, Tang CH, Khodor YL, Vodala S, Menet JS, Rosbash M. 2013. Nascent-seq analysis of *Drosophila* cycling gene expression. Proc Natl Acad Sci 110: E275–E284. 10.1073/pnas.1219969110 23297234 PMC3557077

[GAD350434YUC37] Schlackow M, Nojima T, Gomes T, Dhir A, Carmo-Fonseca M, Proudfoot NJ. 2017. Distinctive patterns of transcription and RNA processing for human lincRNAs. Mol Cell 65: 25–38. 10.1016/j.molcel.2016.11.029 28017589 PMC5222723

[GAD350434YUC38] Smith T, Heger A, Sudbery I. 2017. UMI-tools: modeling sequencing errors in unique molecular identifiers to improve quantification accuracy. Genome Res 27: 491–499. 10.1101/gr.209601.116 28100584 PMC5340976

[GAD350434YUC39] So WV, Rosbash M. 1997. Post-transcriptional regulation contributes to *Drosophila* clock gene mRNA cycling. EMBO J 16: 7146–7155. 10.1093/emboj/16.23.7146 9384591 PMC1170315

[GAD350434YUC40] Sousa-Luís R, Dujardin G, Zukher I, Kimura H, Weldon C, Carmo-Fonseca M, Proudfoot NJ, Nojima T. 2021. POINT technology illuminates the processing of polymerase-associated intact nascent transcripts. Mol Cell 81: 1935–1950.e6. 10.1016/j.molcel.2021.02.034 33735606 PMC8122139

[GAD350434YUC41] Taylor P, Hardin PE. 2008. Rhythmic E-box binding by CLK–CYC controls daily cycles in *per* and *tim* transcription and chromatin modifications. Mol Cell Biol 28: 4642–4652. 10.1128/MCB.01612-07 18474612 PMC2447118

[GAD350434YUC42] Weber CM, Ramachandran S, Henikoff S. 2014. Nucleosomes are context-specific, H2A.Z-modulated barriers to RNA polymerase. Mol Cell 53: 819–830. 10.1016/j.molcel.2014.02.014 24606920

[GAD350434YUC43] Wickham H. 2016. ggplot2: elegant graphics for data analysis. Springer-Verlag, New York.

[GAD350434YUC44] Wissink EM, Vihervaara A, Tippens ND, Lis JT. 2019. Nascent RNA analyses: tracking transcription and its regulation. Nat Rev Genet 20: 705–723. 10.1038/s41576-019-0159-6 31399713 PMC6858503

[GAD350434YUC45] Wu DC, Lambowitz AM. 2017. Facile single-stranded DNA sequencing of human plasma DNA via thermostable group II intron reverse transcriptase template switching. Sci Rep 7: 8421. 10.1038/s41598-017-09064-w 28827600 PMC5566474

[GAD350434YUC46] Wuarin J, Schibler U. 1994. Physical isolation of nascent RNA chains transcribed by RNA polymerase II: evidence for cotranscriptional splicing. Mol Cell Biol 14: 7219–7225. 10.1128/mcb.14.11.7219-7225.1994 7523861 PMC359256

[GAD350434YUC47] Xu H, Nottingham RM, Lambowitz AM. 2021. TGIRT-seq protocol for the comprehensive profiling of coding and non-coding RNA biotypes in cellular, extracellular vesicle, and plasma RNAs. Bio Protoc 11: e4239. 10.21769/BioProtoc.4239 PMC8678547 35005084

[GAD350434YUC48] Yao J, Wu DC, Nottingham RM, Lambowitz AM. 2020. Identification of protein-protected mRNA fragments and structured excised intron RNAs in human plasma by TGIRT-seq peak calling. Elife 9: e60743. 10.7554/eLife.60743 32876046 PMC7518892

[GAD350434YUC49] Zhang Y, Liu T, Meyer CA, Eeckhoute J, Johnson DS, Bernstein BE, Nusbaum C, Myers RM, Brown M, Li W, 2008. Model-based analysis of ChIP-seq (MACS). Genome Biol 9: R137. 10.1186/gb-2008-9-9-r137 18798982 PMC2592715

[GAD350434YUC50] Zubradt M, Gupta P, Persad S, Lambowitz AM, Weissman JS, Rouskin S. 2017. DMS-MaPseq for genome-wide or targeted RNA structure probing in vivo. Nat Methods 14: 75–82. 10.1038/nmeth.4057 27819661 PMC5508988

