# Supplementary material for: Butt-seq: a new method for facile profiling of transcription
Source: Genes Dev. 2023 May 1;37(9-10):432–48. doi: 10.1101/gad.350434.123 (PMC10270195; doi:10.1101/gad.350434.123)
Supplement: Supplemental Material [file supp_37_9-10_432__DC1.html]

Butt-seq: a new method for facile profiling of transcription — Supplemental Material 

# Butt-seq: a new method for facile profiling of transcription

## Supplemental Material

- Supplemental\_Primers.xlsx
- Supplemental\_Detailed\_Protocol.docx
- Supplemental\_Figures.pdf
