# Supplementary material for: Butt-seq: a new method for facile profiling of transcription
Source: Genes Dev. 2023 May 1;37(9-10):432–48. doi: 10.1101/gad.350434.123 (PMC10270195; doi:10.1101/gad.350434.123)
Supplement: Supplemental Material [file supp_gad.350434.123_Supplemental_Detailed_Protocol.docx]

## Reagents:

TGIRT-III (Ingex)

PEG3350 (Sigma, 202444)

HEPES (Sigma, H3375)

TCEP HCl (Goldbio, TCEP1)

NaCl (Sigma, S9888)

MgCl_2_ (ThermoFisher, AM9530G)

dNTPs (ThermoFisher, R0181)

Exonuclease III (NEB, M0206L)

NaOH (Sigma, 79724)

HCl (Sigma, H9892)

Ampure XP (Fisher, NC9933872)

Ethanol, 200 proof (Fisher, 22-032-601)

10% SDS Solution (Invitrogen, 15553027)

NEBNext Ultra II Q5 Master Mix (NEB, M0544L)

Novex 8% TBE Gel, 12-well (Thermofisher, EC62152BOX)

10x TBE Running Buffer (Thermofisher, J62788.K2)

Purple Loading Dye (NEB, B7024S)

SYBR Gold (ThermoFisher, S11494)

Costar Spin-X Centrifuge Filters (Fisher, 07-200-386)

Isopropanol (Sigma, 278475)

Glycoblue (AM9516)

## Buffers:

10x Annealing Buffer

10mM Tris-HCl, pH7.5

10mM EDTA

10mM TCEP pH 7.5

10x ButtRT Buffer

100mM HEPES, pH 8

500mM NaCl

50mM MgCl_2_

10mM TCEP

## Annealing Primers:

1. Prepare the following annealing reactions:

SCR2R RNA/DNA Hybrid Primer:

| Reagent | Volume |
| --- | --- |
| SCR2R RNA primer, 10 µM | 10 µl |
| SCR2 DNA primer, mixed, 10 µM | 10 µl |
| 10x annealing buffer | 5 µl |
| H2O | 25 µl |

MER1R RNA/DNA Hybrid Primer:

| Reagent | Volume |
| --- | --- |
| R1MER RNA primer, 10 µM | 10 µl |
| R1ME DNA primer, mixed, 10 µM | 10 µl |
| 10x annealing buffer | 5 µl |
| H2O | 25 µl |

*Note: R1ME and SCR2 DNA primers consists of a hand-mixed equimolar ratios of primers with A/T/C/G at the end. For sequences, see supplementary primer table.*

*Note: The primer table lists PAGE-purified IDT ultramers for SCR2 DNA primers. We noticed that when not using Ultramer synthesis with an oligo as long as SCR2 length, upwards of 50-70% of the received product would be partially synthesized oligonucleotide.*

1. Prepare the following PCR program, “Anneal Primers”:

| **Temperature** | **Time** |
| --- | --- |
| 88°C | Preheat/Hold |
| 88°C | 2:00 |
| 0.1°C/s | Ramp down |
| 10°C | Hold |

1. Start the Anneal Primers program to preheat the thermal cycler
2. Place RNA/DNA hybrid primers in the preheated thermal cycler and resume the program
3. After reaching 10°C, aliquot into single-use aliquots and store at -80°C for up to six months.

*Note: Freeze-thawing the RNA/DNA hybrid primer leads to loss of performance.*

## Preparing ButtRT Buffer:

1. Add the following reagents to a tube in the specified volumes to make ButtRT Buffer:
   - 100 µL of 1M HEPES, pH 8.0 (to make a final concentration of 100 mM)
   - 20 µL of 0.5M TCEP-NaOH, pH 7.5 (to make a final concentration of 10 mM)
   - 100 µL of 5M NaCl (to make a final concentration of 500 mM)
   - 50 µL of 1M MgCl2 (to make a final concentration of 50 mM)
   - 730 µL of H2O
2. Heat the prepared ButtRT Buffer to 65°C for 10 minutes to inactivate any RNases present in the buffer, which is enabled by the presence of TCEP (Rhee & Burke, 2004; Yu et al., 2021). This step is not necessary if all reagents used are RNase-free.
3. Aliquot 10x ButtRT Buffer into 50 µL aliquots and store at -20°C.

*Note: The original TGIRT protocol uses DTT, which we replaced with TCEP as it worked just as well while exhibiting greater consistency and resilience against freeze-thaws. However, we limit ButtRT buffer to <10 freeze thaws to err on the side of caution.*

## 1st Strand Synthesis:

1. Prepare a master mix in 0.2 mL PCR tubes by adding the following reagents in the specified volumes:

| **Reagent** | **Volume** |
| --- | --- |
| 10x ButtRT buffer | 0.5 µL |
| SCR2R, Annealed | 0.2 µL |
| TGIRT | 0.2 µL |
| 50% PEG3350 | 1 µL |
| RNA | 0.5-2.1 µl |
| H2O | To 4 µL |

*Note: This protocol has been tested on serially diluted RNA inputs between 0.1pg to 50ng. However, when starting with <500,000 cells, the RNA quantification step is typically omitted.*

*Note: We suggest preparing a master mix for at least 3 reactions when using the 5µl reaction presented above, and that no less than 0.5µl be pipetted at a time. If pipetting <1µl of TGIRT, using low retention tips can help.*

1. Incubate the master mix on ice for 30 minutes.
2. While incubating, prepare the first strand NaCl/dNTP mix by adding the following reagents in the specified volumes:

| **Reagent** | **Volume** |
| --- | --- |
| 2M NaCl | 30 µl |
| 5mM dNTPs | 1 µl |

*Note: dNTPs stored at concentrations <10mM have been observed to be prone to hydrolysis. At 5mM, we make fresh aliquots every month.*

1. Also while incubating, if not yet done so, prepare the Butt 1^st^ Strand Synthesis Program on your thermal cycler:

| **Temperature** | **Time** |
| --- | --- |
| 25°C | 1:00 |
| 0.5°C/s | Ramp up |
| 60°C | 0:05 |
| 4°C | Hold |

*Note: We have observed some minor inconsistencies between thermal cyclers that’s abrogated by using a specified ramp rate. Using this relatively slower ramp also appears to increase yield.*

1. After the incubation is complete, add 1 µL of the first strand NaCl/dNTP mix to the reaction, spin down, and flick at least 2 times to mix.

*Note: The purpose of the low concentration of dNTPs and increasing the salt concentration during reverse transcription is to slow down the rate of reverse transcription and limit the length of the cDNA. If done correctly, the final library should be <700bp long.*

*Note: Mixing by pipetting up and down slightly reduces yield. This loss is likely negligible, but why accept losses one can easily avoid?*

1. Place the reaction into a thermal cycler and run the Butt 1st Strand PCR Program.

## Exonuclease III Digestion:

*Note: Exonuclease III is a 3’ -> 5’ exonuclease that also acts on RNA/DNA hybrids. Exonuclease III partially digests unligated primer, which can help reduce the incidence of PCR bubbles if starting with low input material. If starting with >10ng of RNA, this step is optional.*

1. While the program is running, prepare the Exo3 Master Mix by adding the following reagents in the specified volumes:

| **Reagent** | **Volume** |
| --- | --- |
| NEBuffer 1 | 1 µL |
| Exonuclease III | 2 µL |
| H­_2_O | 2 µL |

1. Prepare the Exo3 PCR Program:

| **Temperature** | **Time** |
| --- | --- |
| 37°C | 8:00 |
| 4°C | Hold |

1. After the Butt 1st Strand Program has reached 4°C, remove to ice.
2. Add 5 µL of the Exo3 Master Mix to the reaction, mix using any method you wish.
3. Place in the thermal cycler and run the Exo3 Program.

## Release TGIRT and destroy RNA:

1. Add 3µL of 1N NaOH to the reaction, place in the thermal cycler and run the Alkaline Hydrolysis program:

| **Temperature** | **Time** |
| --- | --- |
| 95°C | 5:00 |
| 10°C | Hold |

*Note: This step serves two purposes. First, it removes TGIRT from the cDNA, which would otherwise interfere with second strand synthesis. Second, it hydrolyzes the RNA, such that it won’t be carried over into downstream reactions.*

1. After the reaction reaches 10°C, remove samples from thermal cycler to room temperature.
2. Add 3µl 1N HCl and mix.

## Bead Purification:

1. To each sample, add 24 µL of 95% EtOH and 24 µL of Ampure XP Beads (warmed to RT for at least 30 minutes).

*Note: Because we’re attempting to precipitate ssDNA as small as 80nt, Ampure needs a little help. The addition of alcohol helps precipitate small ssDNA fragments – see (Fishman & A, 2019). The percentage of ethanol was carefully calibrated to minimize the amount of unligated adapter and maximize the amount of desired cDNA, with a reasonable buffer zone if your ethanol is a bit old and watery. This step can be optimized for sensitive applications, but optimization should not be necessary.*

1. Flick to mix, and spin down briefly to remove liquid from cap but not for long enough to pellet magnetic beads.
2. Incubate at RT for at least 10 minutes.
3. Place samples on a magnet and let stand for ~2 minutes, or until samples clear.
4. Remove and discard supernatant
5. Add 200µl 80% EtOH and let stand for at least 30 seconds
6. Remove and discard supernatant
7. Add 200µl 80% EtOH and let stand for at least 30 seconds
8. Remove and discard supernatant
9. Briefly spin down tubes to gather residual EtOH and place back on magnet
10. Using a P20 tip, remove excess EtOH

*Note: The TGIRT reaction will tolerate a little ethanol carryover, so steps 10-11 are optional, but nevertheless advised.*

1. Remove from magnet and dry at room temperature for 2-3 minutes, or until beads just begin to turn from dark to pale brown.
2. Resuspend in 4.1µl H­_2_O and incubate for at least 2 minutes

*Note: The next reaction uses 4µl of cDNA, which may be difficult to pipette without bead carryover – depending on the magnetic rack used. Pipetting an air-bubble into the bottom of the PCR tube and then aspirating liquid from the air-liquid interface is an option. This technique involves pipetting from the meniscus of the liquid after lifting it with an air bubble, and it mitigates any risk of pipetting up any beads by mistake. Alternatively, you can resuspend in volumes >4µl, though great volumes will dilute your final yield accordingly.*

1. Place samples back on magnet for >2 minutes. Samples can be left eluting or on magnet until ready to be added to 2^nd^ stand synthesis reaction.

***Stopping point***: *Clean cDNA can be stored on ice during the day or at -20C overnight. We have no experience with longer storage. cDNA can be left with the beads during storage.*

## Second-strand synthesis

1. Prepare the 2^nd^ Strand Synthesis reaction:

| **Reagent** | **Volume** |
| --- | --- |
| 10x ButtRT buffer | 1 µL |
| meR1R, Annealed | 0.3 µL |
| TGIRT | 0.3 µL |
| 50% PEG3350 | 2 µL |
| H_2_O | 0.4 µl |

1. Add 4µl 2^nd^ Strand Synthesis reaction to a fresh PCR strip tube on ice and add 4µl of the purified cDNA from the previous section.
2. Incubate on ice for 30 minutes
3. While incubating, prepare second strand NaCl/dNTP mix:

| **Reagent** | **Volume** |
| --- | --- |
| 2M NaCl | 20 µl |
| 20mM dNTPs | 1 µl |

*Note: A higher concentration of dNTPs and a longer reverse transcription are used here to maximize the amount of product, as limiting the length is no longer a concern. The salt concentration and reverse transcription duration are nevertheless calibrated to minimize undesirable template switching events.*

1. Also while incubating, if not yet done so, prepare the Butt 2^nd^ Strand PCR program:

| **Temperature** | **Time** |
| --- | --- |
| 25°C | 1:00 |
| 0.5°C/s | Ramp up |
| 60°C | 1:00 |
| 4°C | Hold |

1. Add 2µl dNTPs/NaCl mix to the reaction, flick and spin down twice to mix as above.
2. Place reaction into thermal cycler and run Butt 2^nd^ Strand PCR program. A heated lid is not necessary.
3. After reaction has reached 4C, remove reaction and place on ice.
4. Add 1µl 0.2% SDS to each sample and incubate using the SDSDenature Thermal Cycler program:

| **Temperature** | **Time** |
| --- | --- |
| 55°C | 5:00 |
| 10°C | Hold |

*Note: As before, TGIRT needs to be removed from the DNA product prior to the next step. However, as no RNA needs to be hydrolyzed this time, this simpler reaction with SDS has proved sufficient.*

1. Add 25µl Ampure XP beads (2.27x) to each sample and incubate at room temperature for at least 5 minutes.
2. Place reaction on magnet for 2 minutes or until solution clears.
3. Remove and discard supernatant
4. Add 200µl 80% EtOH and let stand for at least 30 seconds
5. Remove and discard supernatant
6. Add 200µl 80% EtOH and let stand for at least 30 seconds
7. Remove and discard supernatant
8. Spin down PCR tubes briefly and return to magnet
9. Using a P20 tip, remove excess EtOH from the bottom of the tube

*Note: Like before, the next reaction can tolerate a little EtOH carryover, but this step is still advised.*

1. Let dry for 2 minutes, or until beads just begin to turn from dark to light brown.
2. Resuspend beads in 9.1µl H_2_O and incubate for at least 2 minutes

*Note*: *Like before, resuspend in whatever volume you are comfortable eluting from without carryover.*

1. Place on magnet for at least 2 minutes.

***Stopping point:*** *double stranded cDNA (ds-cDNA) can be stored on ice for a day or at -20C for up to overnight. We have no experience with longer storage durations.*

## PCR

1. Prepare PCR Reactions:

| **Reagent** | **Volume** |
| --- | --- |
| NEBNext Ultra II Q5 2x PCR Master Mix | 25 µL |
| 10µM SCP7 | 2.5 µL |
| 10µM Ad1.XX | 2.5 µL |
| ds-cDNA | 9 µl |
| H2O | 11 µL |

*Note: Ad1.xx is a library-specific barcode. Be sure to spin down your unique barcode primers before opening them, as barcode cross-contamination is potentially disastrous.*

*Note: The master mix is dispensed at 38.5µl. That number is provided here for your convenience. The reason we do not elute the ds-cDNA at 20µl and include no H_2_O in the master mix is because the liquid properties of the mastermix cause it to be overpipetted if the 2x is not sufficiently dilute. This is not critical, but it is annoying.*

1. Run samples on the ButtQ5 PCR reaction:

| **Temperature** | **Time** |
| --- | --- |
| 72°C* | 2:00 |
| 98°C | 2:00 |
| 98°C | 0:10 |
| 65°C | 0:10 |
| Repeat 3-4 | 8-15x |
| 72°C | 1:00 |

**The initial 72*°*C incubation is to fill in the single-stranded meR1R adapter, effectively doubling your starting product.*

*Note: Overamplification and PCR bubbles are a serious concern in Butt-Seq, as it will lead to excessive adapter contamination in the final library. For initial experiments, we suggest optimizing by separating the ds-cDNA into 3 PCR reactions with 9, 11, and 13 cycles.*

*Note: Omitting the Exonuclease III digestion or using >13 PCR cycles may make “Recondioned PCR” desirable. See below.*

1. Add 65µl Ampure XP beads (1.3x) to PCR reaction and incubate for at least 5 minutes.
2. Place reactions on magnet and incubate for >2 minutes or until supernatant is clear.
3. Remove and discard supernatant.
4. Add 200µl 80% EtOH and incubate for at least 30 seconds
5. Remove and discard supernatant.
6. Add 200µl 80% EtOH and incubate for at least 30 seconds.
7. Remove and discard supernatant.
8. Spin down briefly and place back on magnet
9. Using a P20 tip, remove excess EtOH

*Note: This step was optional in previous cleanups, but it is not optional here. If you do not remove the excess EtOH here, your samples will float out of your gel.*

1. Remove from magnet and let dry at room temperature for 2-3 minutes, or until samples just begin to turn from dark to light brown.
2. Resuspend in 10µl 1x Purple Loading Dye and incubate for at least 2 minutes.
   1. If doing reconditioning PCR, resuspend in 9.1µl H_2_O
3. Place on magnet for at least 2 minutes or until supernatant clears.

***STOPPING POINT****: Clean PCR product, with or without beads, can be stored for quite a while at -20C. We have stored for up to a week. If freezing product in purple loading dye, we suggest remixing the samples prior to loading by flicking or pipetting up and down, as we have occasionally witnessed separation causing samples to float out of the gel.*

1. If >13 cycles were used, or >11 cycles if Exonuclease III treatment was omitted, proceed to the next section: “Reconditioning PCR”. Otherwise, skip to Section titled “Gel Extraction”

## OPTIONAL: Reconditioning PCR

This step is recommended if >13 cycles are used, or >11 cycles if Exonuclease III treatment is omitted. PCR bubbles of your desired library will interfere with quantification, but adapters with a few bases of non-template addition can also form bubbles that will contaminate your final library. See (Thompson et al., 2002) for more information, or Google “reconditioning PCR” for many anecdotal experiences. Reconditioning PCR is not used in our typical workflow, and we encourage users to optimize PCR cycles to avoid it. No reconditioned libraries were used in the parent manuscript.

We present this option here to enable users to rescue valuable libraries that have gone awry.

This step can be used before or after gel extraction, but if conducted after gel extraction, extra cycles and a second gel extraction are required.

1. Prepare PCR Reactions:

| **Reagent** | **Volume** |
| --- | --- |
| NEBNext Ultra II Q5 2x PCR Master Mix | 25 µL |
| 10µM SCP7 | 2.5 µL |
| 10µM Ad1.XX | 2.5 µL |
| PCR Product | 9 µl |
| H2O | 11 µL |

*Note: Make sure your Ad1.XX matches the one you used before!*

1. Run samples with the following ButtQ5-Recondition PCR program:

| **Temperature** | **Time** |
| --- | --- |
| 72°C* | 2:00 |
| 98°C | 2:00 |
| 98°C | 0:10 |
| 65°C | 0:10 |
| Repeat 3-4* | 1x or 3x |
| 72°C | 1:00 |

*Note: 1x if going straight from the previous step, 3x if libraries have already been gel extracted once to compensate for loss of material from gel extraction.*

1. Add 65µl Ampure XP beads (1.3x) to PCR reaction and incubate for at least 5 minutes.
2. Place reactions on magnet and incubate for >2 minutes or until supernatant is clear.
3. Remove and discard supernatant.
4. Add 200µl 80% EtOH and incubate for at least 30 seconds.
5. Remove and discard supernatant.
6. Add 200µl 80% EtOH and incubate for at least 30 seconds.
7. Remove and discard supernatant.
8. Spin down briefly and place back on magnet.
9. Using a P20 tip, remove excess EtOH.

*Note: This step was optional in previous cleanups, but it is not optional here. If you don’t remove the excess EtOH here, your samples will float out of your gel.*

1. Remove from magnet and let dry at room temperature for 2-3 minutes, or until samples just begin to turn from dark to light brown.
2. Resuspend in 10µl 1x Purple Loading Dye and incubate for at least 2 minutes.
   1. If doing reconditioning PCR, resuspend in 9.1µl H_2_O
3. Place on magnet for at least 2 minutes or until supernatant clears.

***STOPPING POINT****: Clean PCR product, with or without beads, can be stored for quite a while at -20C. We have stored for up to a week. If freezing product in purple loading dye, we suggest remixing the samples prior to loading by flicking or pipetting up and down, as we have occasionally witnessed separation causing samples to float out of the gel.*

## Gel Extraction

1. Load samples on an 8% TBE Gel, using 0.5µl NEB TriDye Ultra Low Range DNA ladder diluted in 10µl 1x Purple Loading Dye as a ladder. If your samples float, you have EtOH carryover.
2. Run at 180V for 45 minutes.
3. Remove gel from cassette and stain with 1x Sybr Gold for 5 minutes.
4. While staining, prepare one 0.5ml tube per library.
5. Image the gel on a blue light transilluminator.

*Note: As long as the smear is even faintly visible, usable library should be produced. Even if the smear is only faintly visible at smaller sizes, always excise the region up to 700bp.*

1. Excise the smear ranging from 150bp to 700bp and remove the slice into the 0.5ml tube.

*Note*: *Adapters are approximately 140bp, so excising at 150bp will give the greatest breadth of pause sites but may include a large proportion of unmappable fragments. Typical NET-Seq protocols excise inserts >25-35bp in length, so the excised region may be adjusted at the experimentalist’s discretion.*

## Example gel results:


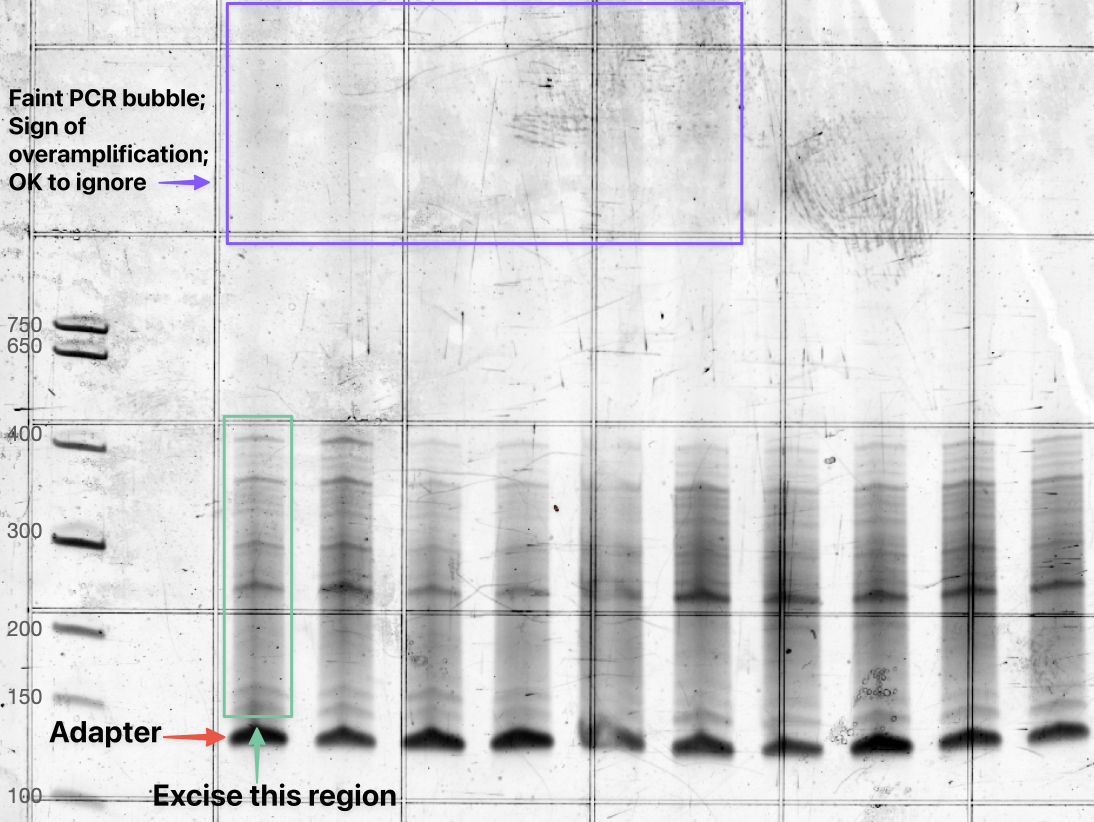


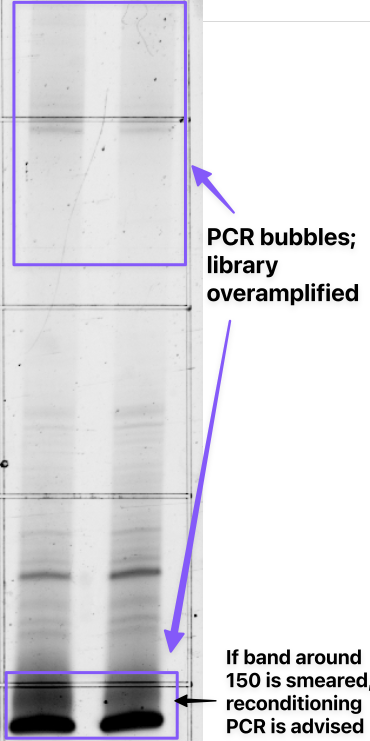


1. Poke a hole in the bottom of the 0.5ml tube with a 22 gauge needle
2. Nest the 0.5ml tube inside a 2.0ml tube.
3. Spin at >18,000 RCF for 2 minutes

*Note: If gel fragments remain in the 0.5ml tube after spinning, poke a second hole and spin it again for 1 minute.*

1. Add 600µl 0.3M NaCl to gel fragments and incubate at 75C for 15 minutes, flicking to mix every 3-5 minutes.

*Note: If you have a Thermomixer or an equivalent, you can shake it at max speed for those 15 minutes instead*

*Alternative: Incubate overnight at 4C.*

1. Using a wide-bore P1000 tip or a P1000 tip with the end cut off, transfer gel slurry into an 0.22µM Co-Star X column.

*Note: The purpose of this step is to filter out gel fragments, so it is not necessary to transfer all the gel fragments. In fact, it is preferable not to transfer them, to reduce changes of clogging the filter.*

1. Spin at >18,000 RCF for 2 minutes

*Note: If any liquid remains in the column, stir fragments with a P1000 tip and spin for another minute.*

1. Add 600µl Isopropanol and 1µl Glycoblue.
2. Incubate for at least 10 minutes at room temperature or between 1 hour to overnight at -20°C.

*Note: Although the longer incubation at -20*°*C does increase yield, this larger yield is typically unnecessary because this is a post-PCR step. If your library was barely visible in the gel, an overnight incubation is advised.*

1. Spin for 20 minutes at 4°C at >18,000 RCF

*Note: Again, longer spins increase yield 5-10% (Li et al., 2020). The gain is usually negligible.*

1. Remove supernatant by pipetting or decanting; blue pellet should be visible at the bottom of the tube.
2. Add 80% EtOH, invert 5 times to wash, and spin down for 5 minutes at >18,000 RCF. It is not necessary to dislodge the pellet if it is stuck.
3. Remove EtOH by pipetting or decanting.
4. Add 80% EtOH, invert 5 times to wash, and spin down for 5 minutes at >18,000 RCF.
5. Remove EtOH by pipetting or decanting.
6. Spin down to gather residual EtOH and remove with a P20 pipette. If >20µl EtOH remains, spin down again and repeat.
7. Let air dry for 10 minutes.
8. Resuspend in 5-20µl H_2_O.

*Note: Typically, we resuspend in 10µl H_2_O. If the library was especially faint on the gel, 5-7µl can be used to recover a higher concentration library.*

1. Proceed with Tapestation D1000 analysis. An ideal tapestation trace is presented below:


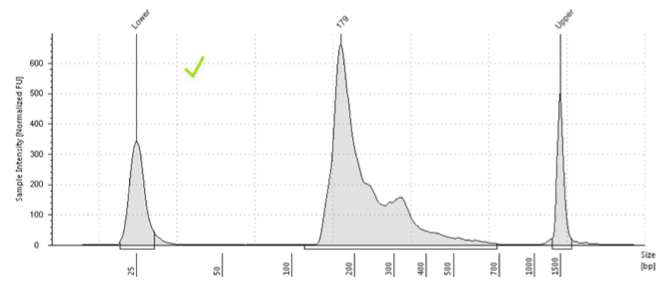


When running an electronic ladder on the D1000 tape, the region between 170-190bp corresponds to around ~160bp, indicating a ~20nt long insert. Although the 180bp peak is usually the highest, occasionally, we get traces that look like this:


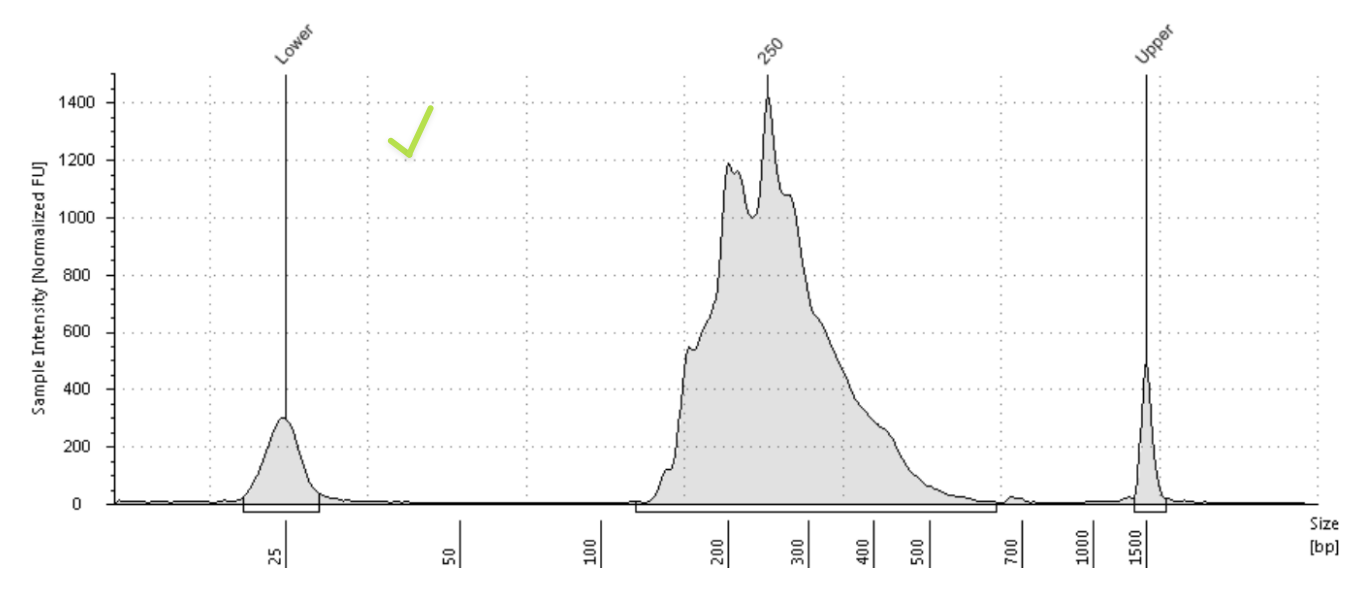


Though the 180bp is not the highest, it is still present, and we still get good results from libraries that look like this. Note that this library also features some minor, acceptable adaptor dimer contamination at ~150.

Concern begins to arise if your libraries look like this:


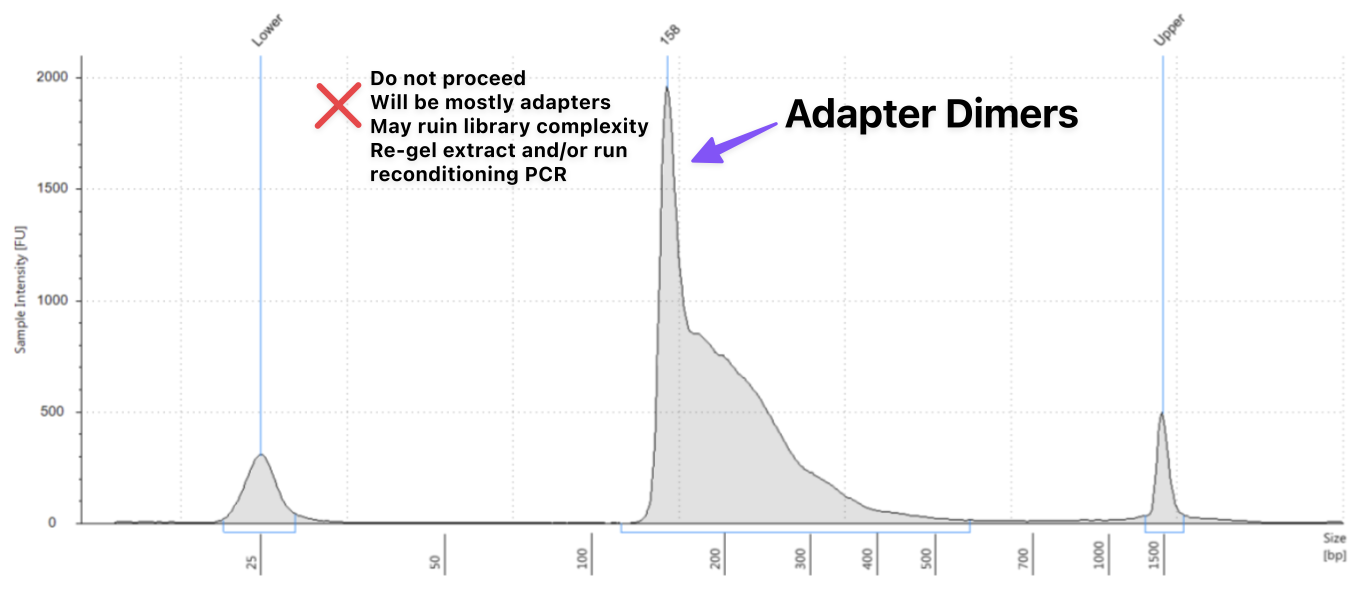


This library is heavily contaminated with adapter dimers, indicative of extreme PCR bubbling or cutting too low on the gel. Re-extract or run reconditioning PCR and re-extract.

1. Quantify libraries. We usually quantify by measuring peak quantifications in the Tapestation software by centering the peak at the ~180bp peak but extending the quantified area to cover the entire library. Region quantification will underestimate the library concentration. If greater precision is desired, one can use qPCR to quantify their libraries and assume a library size of 180bp.
2. Sequence libraries. We usually run Butt-Seq libraries on a 75-cycle kit on Nextseq 500. When loading 20pm of library, we usually load 1-1.5pm of library – corresponding to 20 million reads. If sequencing an organism other than Drosophila, please scale up accordingly.
3. Demultiplex reads. To demultiplex, edit the following line in RunInfo.xml:

<Read Number="2" NumCycles="8" IsIndexedRead="Y" />

To

<Read Number="2" NumCycles="8" IsIndexedRead="N" />

After changing RunInfo.xml, demultiplex with Bcl2fastq as usual.

1. By default, when demultiplexing Nextseq reads, Bcl2fastq will output 3 files that following this naming format: _R#_001.fastq.gz. If your files are named differently, and you plan on using my snakemake pipeline, please change them to match this format.
2. Process the data using the Snakemake script at <https://github.com/albertdyu/BuTTSeq>

Fishman, A., & A, T. L. (2019). QsRNA-seq: A protocol for generating libraries for high-throughput sequencing of small RNAs. *Bio Protoc*, *9*(5), e3179. <https://doi.org/10.21769/BioProtoc.3179>

Li, Y., Chen, S., Liu, N., Ma, L., Wang, T., Veedu, R. N., Li, T., Zhang, F., Zhou, H., Cheng, X., & Jing, X. (2020). A systematic investigation of key factors of nucleic acid precipitation toward optimized DNA/RNA isolation. *Biotechniques*, *68*(4), 191-199. <https://doi.org/10.2144/btn-2019-0109>

Rhee, S. S., & Burke, D. H. (2004). Tris(2-carboxyethyl)phosphine stabilization of RNA: comparison with dithiothreitol for use with nucleic acid and thiophosphoryl chemistry. *Anal Biochem*, *325*(1), 137-143. <https://doi.org/10.1016/j.ab.2003.10.019>

Thompson, J. R., Marcelino, L. A., & Polz, M. F. (2002). Heteroduplexes in mixed-template amplifications: formation, consequence and elimination by 'reconditioning PCR'. *Nucleic Acids Res*, *30*(9), 2083-2088. <https://doi.org/10.1093/nar/30.9.2083>

Yu, A. D., Galatsis, K., Zheng, J., Le, J. Q., Ma, D., Perlman, S., & Rosbash, M. (2021). Development of a Saliva-Optimized RT-LAMP Assay for SARS-CoV-2. *J Biomol Tech*, *32*(3), 102-113. <https://doi.org/10.7171/jbt.21-3203-005>
