# Supplementary material for: Butt-seq: a new method for facile profiling of transcription
Source: Genes Dev. 2023 May 1;37(9-10):432–48. doi: 10.1101/gad.350434.123 (PMC10270195; doi:10.1101/gad.350434.123)
Supplement: Supplemental Material [file supp_gad.350434.123_SupplementaryFigures.pdf]

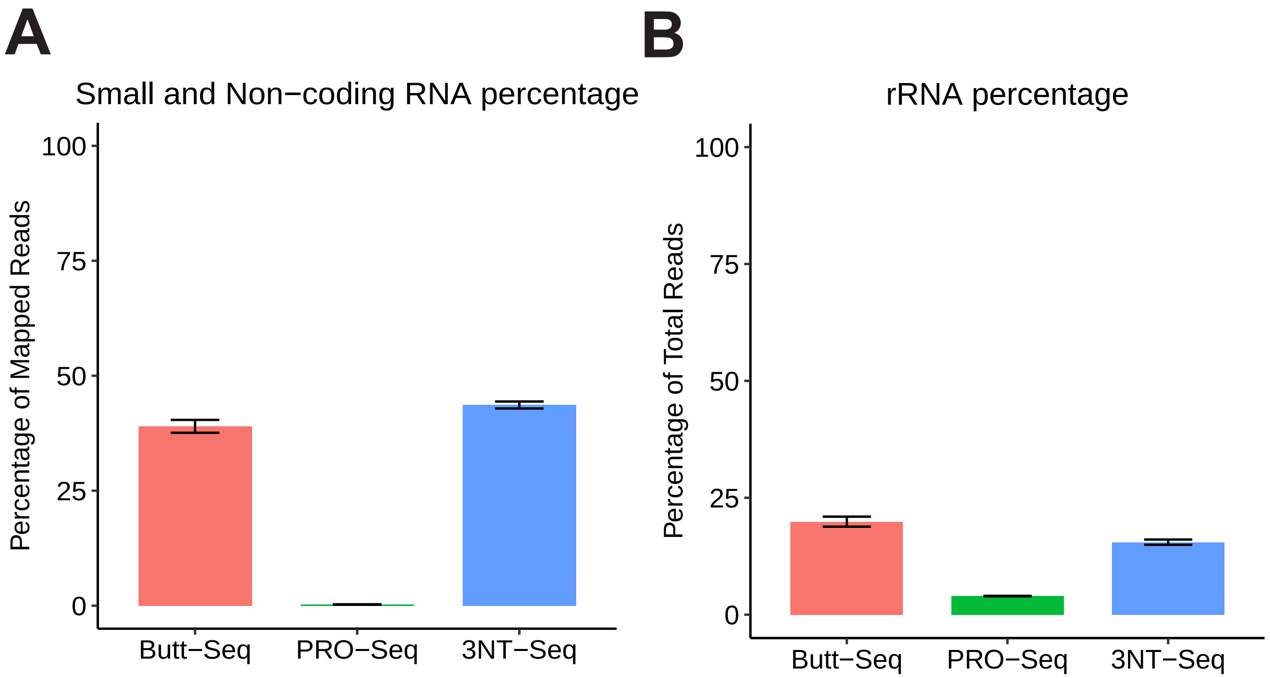

Supplementary Figure 1. Contaminant measures in different techniques. (A) Percentage of mapped reads that map to snoRNAs, snRNAs, and scaRNAs. (B) Percentage of total reads that map to a custom genome annotation containing only rRNA.

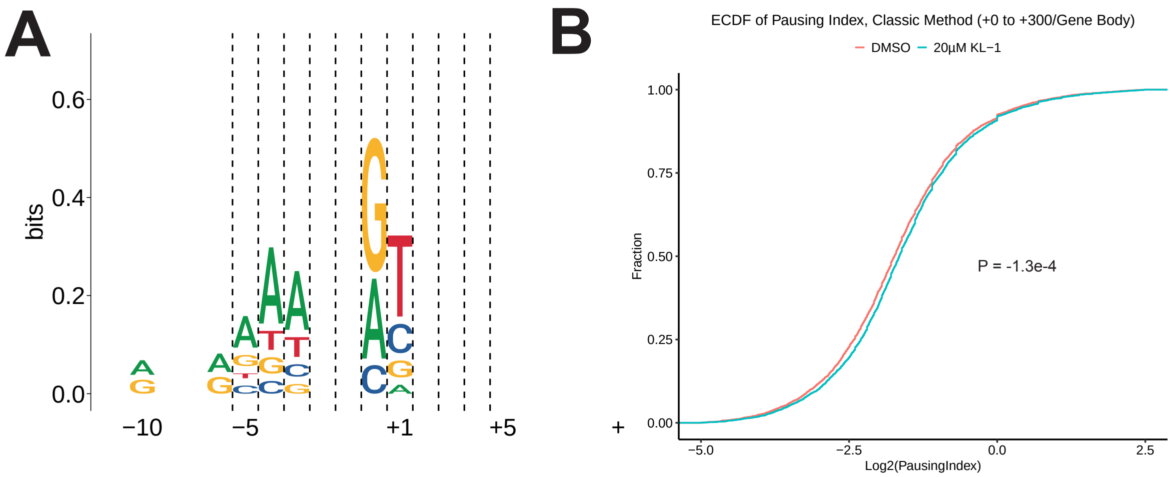

Supplementary Figure 2. Butt-Seq recapitulates known features of pausing. (A) Motif identified from Butt-Seq pauses located within 200nt downstream of the TSS identified by PDA. (B) ECDF of pausing index in Butt-Seq in S2 cells treated with DMSO or 20μM KL-1. Pausing index here is defined as -50 to +200 around the TSS divided by signal across the gene body.
